# Cell-surface localization of Pellino antagonizes Toll-mediated innate immune signalling by controlling MyD88 turnover in *Drosophila*

**DOI:** 10.1038/ncomms4458

**Published:** 2014-03-17

**Authors:** Shanming Ji, Ming Sun, Xiudeng Zheng, Lin Li, Liwei Sun, Dahua Chen, Qinmiao Sun

**Affiliations:** 1 State Key Laboratory of Biomembrane and Membrane Biotechnology, Institute of Zoology, Chinese Academy of Sciences, Beijing 100101, China; 2 State Key Laboratory of Reproductive Biology, Institute of Zoology, Chinese Academy of Sciences, Beijing 100101, China; 3 Centre for Computational and Evolutionary Biology, Institute of Zoology, Chinese Academy of Sciences, 1 Beichen West Road, Chaoyang District, Beijing 100101, China; 4 These authors contributed equally to this work

## Abstract

Innate immunity mediated by Toll signalling has been extensively studied, but how Toll signalling is precisely controlled in balancing innate immune responses remains poorly understood. It was reported that the plasma membrane localization of *Drosophila* MyD88 is necessary for the recruitment of cytosolic adaptor Tube to the cell surface, thus contributing to Toll signalling transduction. Here we demonstrate that *Drosophila* Pellino functions as a negative regulator in Toll-mediated signalling. We show that Pellino accumulates at the plasma membrane upon the activation of Toll signalling in a MyD88-dependent manner. Moreover, we find that Pellino is associated with MyD88 via its CTE domain, which is necessary and sufficient to promote Pellino accumulation at the plasma membrane where it targets MyD88 for ubiquitination and degradation. Collectively, our study uncovers a mechanism by which a feedback regulatory loop involving MyD88 and Pellino controls Toll-mediated signalling, thereby maintaining homeostasis of host innate immunity.

## Abstract (web summary)

Toll signalling activates the innate immune response; however, it remains unclear how this pathway is suppressed to avoid excessive inflammatory responses. Here, the authors report that Pellino, a RING domain-containing ubiquitin E3 ligase, is a negative regulator of Toll signalling in Drosophila.

## Graphical abstract

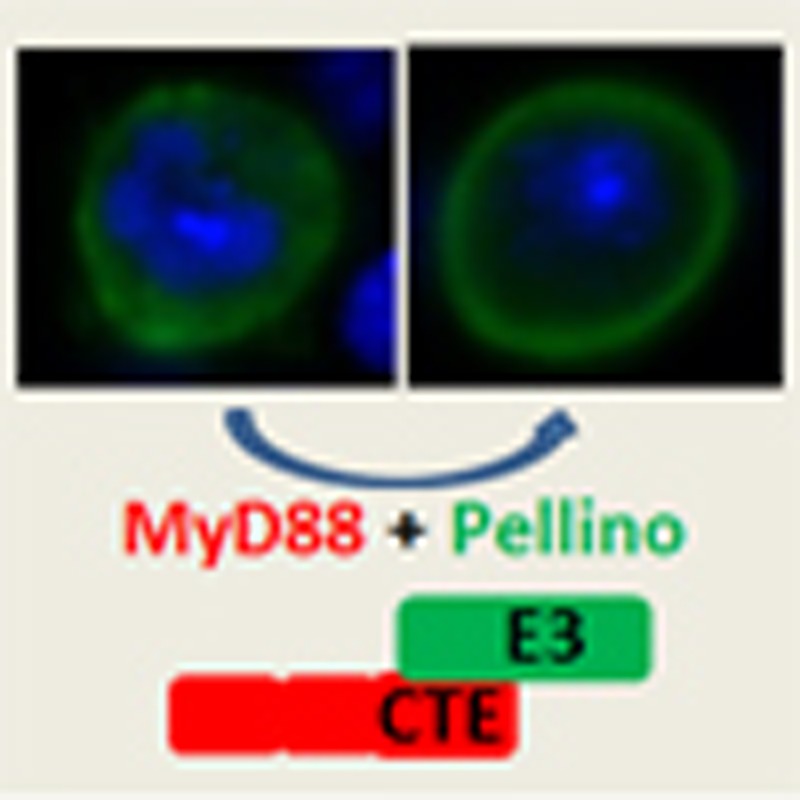

Innate immunity is the first line of host defence against invading microorganisms, including bacteria, fungi and viruses^1^. Understanding the mechanisms of innate immune signalling regulation is critical in immunology. *Drosophila* has provided an ideal model to study the genetic regulation of innate immunity, given its powerful *in vivo* genetics^2^,^3^,^4^. In *Drosophila*, the Toll and the immune deficiency (IMD) pathways are involved in innate immune responses by controlling the transcriptional expression of genes encoding antimicrobial peptides, and both pathways are evolutionarily conserved in mammals^3^,^4^. While the Toll pathway is highly analogous to the interleukin-1 receptor (IL-1R) and MyD88-dependent TLR pathways in mammals, the regulation of IMD pathway is similar to the tumour necrosis factor receptor pathway^5^,^6^.

In the IMD pathway, Gram-negative bacterial infection results in the expression of genes encoding antimicrobial peptides such as Attacin, Cecropin and Diptericin^7^,^8^. Expression of these antimicrobial genes requires the signal-dependent cleavage and subsequent nuclear translocation of Relish, a member of the NF-κB family of transcription factors^7^,^8^. In contrast, in the Toll pathway Spaetzle is proteolytically activated by a serine protease cascade in the circulatory system and binds to the transmembrane receptor Toll upon Gram-positive bacteria or fungal infection^9^,^10^,^11^. The subsequent intracellular cascade results in the dissociation of the NF-κB family member Dorsal or Dif (Dorsal-related immunity factor) from its inhibitor Cactus, the *Drosophila* homologue of IκB-like protein, through the recruitment of the myeloid differentiation factor 88 homologue (MyD88)^12^,^13^, and the *Drosophila* IL-1R-associated kinases, Pelle and Tube (*Drosophila* IRAK-1 and IRAK-4 homologues). Activated Dif thus accumulates in the nucleus and subsequently promotes the expression of downstream target genes in adult flies, such as *drosomycin* and *metchnikowin*^10^,^14^.

Plasma membrane localization of receptors and their associated adaptors has been proposed to be important for signalling transduction, although internalized receptors through endosomes could also promote signalling activity^15^,^16^. In the *Drosophila* Toll signalling pathway, besides the Toll receptor, receptor-associated adaptor MyD88 is also localized on the plasma membrane through its C-terminal domain in a phosphoinositide-binding-dependent manner^17^,^18^. It was recently reported that plasma membrane localization is necessary and sufficient for MyD88 to mediate Toll signalling activity^17^, emphasizing the importance of plasma membrane localization of the Toll receptor and MyD88 for signalling transduction. However, how the signalling activity through plasma membrane localization is regulated remains elusive.

Pellino was initially identified as a Pelle interacting protein in *Drosophila*^19^. In mammals, three members of the Pellino family have been characterized, including Pellino1, Pellino2 and Pellino3, which were previously shown to function as ubiquitin E3 ligases to regulate TLR-mediated innate immune responses^20^,^21^,^22^,^23^,^24^. In contrast, the *Drosophila* genome harbours only one *pellino* gene that encodes *Drosophila* Pellino protein. It was reported that the *Drosophila* Pellino functions as a positive regulator in the *Drosophila* Toll-mediated innate immune pathway^25^; however, the molecular basis of the action of *Drosophila* Pellino is unknown.

In an effort to investigate the potential role of the RING-containing ubiquitin E3 ligases in the innate immunity pathway, we performed a cell-based luciferase reporter screen. Surprisingly, we found that the *Drosophila* Pellino acts as a negative regulator in both the S2 cell culture and the *in vivo* system to control *Drosophila* Toll-mediated innate immune pathway. Pellino accumulates at the plasma membrane upon Toll pathway activation in a MyD88-dependent manner. Moreover, Pellino associates with MyD88 via its CTE domain (a phosphoinositide-binding domain at the C-terminal of MyD88) to target MyD88 for ubiquitination and degradation. Thus, our study unravels a novel feedback regulatory mechanism involving MyD88 and Pellino that contributes to robustness of Toll signalling transduction.

## Results

### Pellino is a potential negative regulator in Toll signalling

**Figure 1:**
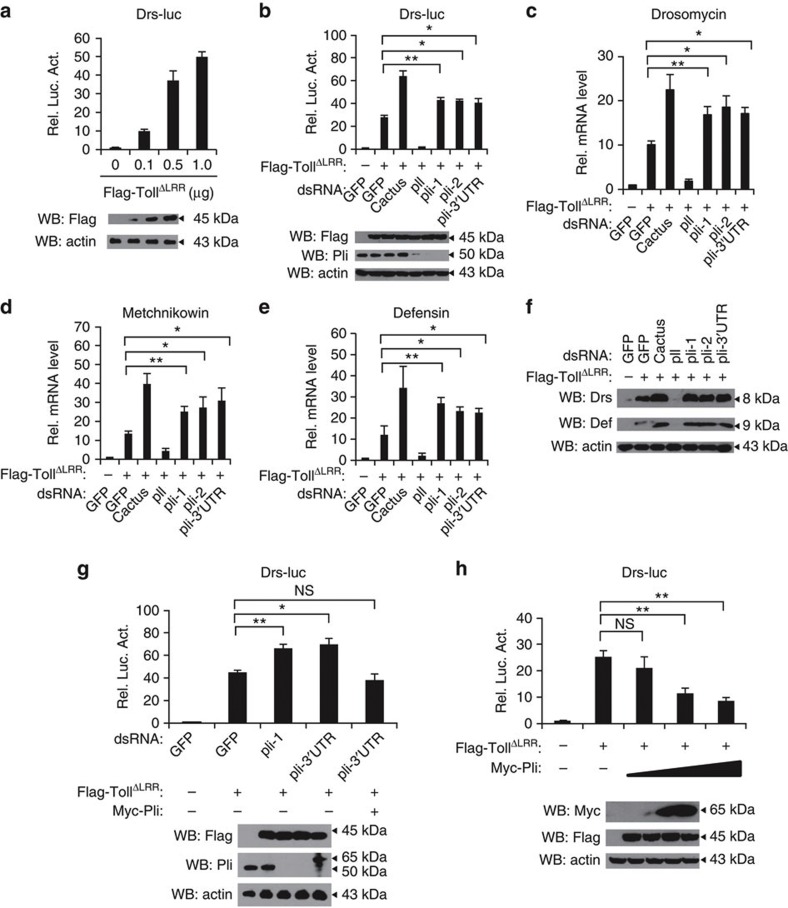
Pellino negatively regulates Toll signalling in *Drosophila*. (**a**) S2 cells were transfected with the expression empty or different doses of Toll^ΔLRR^ vectors together with Drs-luc and Renilla-luc plasmids. Thirty-six hours after transfection, cells were lysed for luciferase assays (upper panel) and immunoblotting assays (lower panel). Luciferase activity was measured and normalized based on renilla luciferase activity. Error bars represent s.d. (*n*=3). (**b**) DsRNAs targeting *gfp*, *cactus* (negative control), *pelle* (*pll*, positive control) or three different regions of *pellino* were treated in S2 cells for 48 h, then the empty vector or Toll^ΔLRR^ plasmids were transfected in S2 RNAi cells with Drs-luc and Renilla-luc plasmids. At 36 h post-transfection, cells were lysed for luciferase assays (upper panel), and immunoblotting with a Pellino-specific antibody to confirm RNAi efficiency (lower panel). Error bars represent s.d. (*n*=3). (**c**–**e**) S2 cells were treated with dsRNAs targeting *gfp*, *cactus*, *pelle* or *pellino*. After 48 h treatment, cells were transfected with DNA vector expressing Toll^ΔLRR^. At 36 h post-transfection, total RNA was isolated for quantitative RT-PCR to determine transcriptional levels of *drosomycin* (**c**) *metchnikowin* (**d**) and *defensin* (**e**). Error bars represent s.d. (*n*=3). (**f**) S2 cells were pretreated with *gfp*, *cactus*, *pelle* and *pellino* dsRNAs for 48 h and then were transfected with vectors expressing Toll^ΔLRR^. Forty-eight hours after transfection, cells were lysed for western blot assays to measure protein levels of Drosomycin and Defensin. Actin is shown as loading control. (**g**) S2 cells were treated with dsRNA (as indicated) for 48 h, reporter plasmids and the indicated expression vectors were transfected into dsRNA knockdown cells. After 36 h, cells were lysed for luciferase assays (upper panel), and immunoblotting assays (lower panel). Error bars represent s.d. (*n*=3). (**h**) S2 cells were transfected with vector or expression constructs as indicated. Thirty-six hours after transfection, cells were extracted for reporter assays (upper panel) and immunoblotting assays performed with the indicated antibodies (lower panel). Error bars represent s.d. (*n*=3). For data from (**b**–**e**, **g**,**h**), the two-tailed Student’s *t*-test was used to analyse statistical significance. *P<0.05, **P<0.01, NS, no significance, versus control groups.

To understand how the ubiquitination pathway regulates Toll-mediated innate immunity signalling, we employed the *Drosophila* S2 cell system and performed a cell-based RNAi screen to identify novel ubiquitin E3 ligases involved in Toll-mediated innate immunity. The firefly *luciferase* gene was placed under the control of the *drosomycin* promoter^26^. Activation of the Toll pathway by expression of the active form of Toll receptor^11^,^27^, Toll^ΔLRR^, resulted in activation of luciferase expression controlled by the *drosomycin* promoter (Drs-luc) in both dose- and time-dependent manners (Fig. 1a and Supplementary Fig. 1a). Consistent with the results obtained from the luciferase reporter assays, Toll^ΔLRR^ also increased expression levels of endogenous *drosomycin* and *metchnikowin* mRNAs (Supplementary Fig. 1b,c). The dsRNA library used in this screen contained 134 dsRNAs targeting most known and predicted genes encoding *Drosophila* RING-containing ubiquitin E3 ligases (RING-E3s). RING-E3s, collectively representing the large majority of E3s, have been linked to the control of numerous cellular processes, such as DNA repair, cell cycle control and host defence^28^,^29^. The library was analysed to identify candidate genes that affected the reporter expression of Drs-luc. Among the 134 specific dsRNAs designed against *Drosophila* RING-E3s, treatment with one dsRNA targeting *CG5212*, which encodes *Drosophila* Pellino, resulted in a significant upregulation of luciferase activity driven by the *drosomycin* promoter, compared with control dsRNA treatment (Supplementary Fig. 2). This suggested a potential negative role of Pellino in regulating *Drosophila* Toll-mediated innate immunity.

### Pellino has a negative role in Toll-mediated innate immunity

Mammalian Pellino proteins, including three members (Pellino1, Pellino2 and Pellino3), regulate the TLR-mediated innate immune responses^21^,^22^,^23^,^24^. In contrast, the *Drosophila* genome harbours only one *pellino* gene that has been proposed previously to function as a positive regulator in *Drosophila* Toll-mediated innate immune pathway^25^. Because of this inconsistency, we characterized the exact role of *Drosophila* Pellino, and sought to understand the molecular mechanism underlying its action in the *Drosophila* innate immunity pathway.

We designed three *pellino* dsRNAs independent of the dsRNA used in the RNAi screen. As shown in a western blot assay, these dsRNAs targeting to two non-overlapping regions of the ORF and the 3′ UTR of the *pellino* gene efficiently knocked down Pellino expression in S2 cultured cells (Fig. 1b). Using the Drs-luc reporter assay, we found that knockdown of *pellino* significantly enhanced the Toll signalling activity stimulated by overexpression of the active form of Toll receptor compared with controls (Fig. 1b). We next examined the expression of antimicrobial peptide genes in S2 cells by performing quantitative RT-PCR (qRT-PCR) assays. In agreement with the results obtained from the luciferase reporter assays, expression levels of endogenous *drosomycin*, *metchnikowin* and *defensin* mRNAs also increased when *pellino* was knocked down in the Toll^ΔLRR^ overexpressing S2 cells, compared with the control (Fig. 1c–e). Consistent results were obtained when Drosomycin and Defensin proteins were measured (Fig. 1f). In addition, rescue experiments showed that overexpression of Pellino appeared to reduce the increased activity of the *drosomycin* promoter by a *pellino* dsRNA that targeted the 3′ UTR of the *pellino* gene (Fig. 1g). Consistent with the above results, overexpression of Pellino inhibited luciferase activity by the *drosomycin* promoter induced by Toll^ΔLRR^ in a dose-dependent manner (Fig. 1h). These findings suggest that Pellino has a negative role in the Toll-mediated innate immune pathway in cultured cells.

### Pellino is dispensable for the IMD pathway

**Figure 2:**
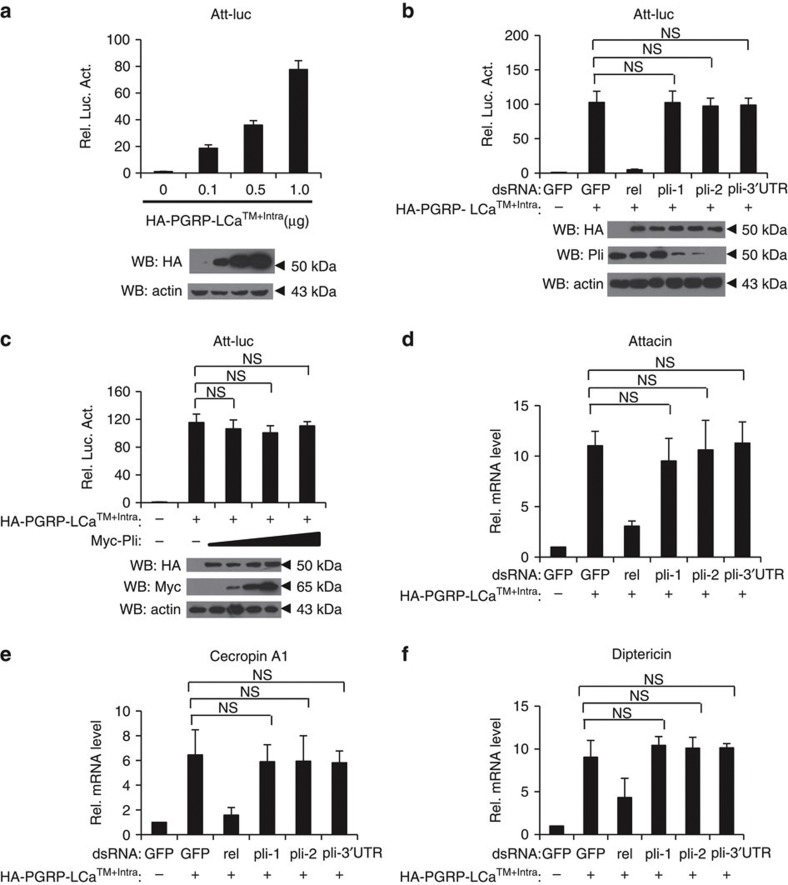
Pellino is not involved in the *Drosophila* IMD pathway. (**a**) S2 cells were transiently transfected with an expression empty vector or increased doses of the PGRP-LCa^TM+Intra^ (PGRP-LCa active form) vectors together with a luciferase reporter construct driven by the promoter of *attacin* gene (Att-luc). Renilla luciferase plasmid was used as an internal control. Thirty-six hours after transfection, luciferase activity was measured and normalized based on renilla luciferase activity (upper panel), and immunoblotting assays were performed (lower panel). Error bars represent s.d. (*n*=3). (**b**) DsRNAs targeting *gfp*, *relish* (*rel*, positive control) or three different regions of *pellino* were used to treat S2 cells for 48 h, then empty vector or HA-PGRP-LCa^TM+Intra^ plasmids were transfected in dsRNA-treated S2 cells with Att-luc and Renilla-luc plasmids. At 36 h post-transfection cells were lysed for luciferase assays (upper panel) and immunoblotting performed to confirm RNAi efficiency (lower panel). Error bars represent s.d. (*n*=3). (**c**) DNA vector expressing PGRP-LCa^TM+Intra^ and increasing amounts of expression Myc-Pellino constructs were transfected into S2 cells with Att-luc and Renilla-luc plasmids. At 36 h post-transfection, cells were lysed for luciferase assay (upper panel), and immunoblotting to check protein expression levels (lower panel). Error bars represent s.d. (*n*=3). (**d**–**f**) S2 cells were treated with dsRNAs targeting *pellino, relish* or *gfp*. After 48 h treatment, cells were transfected with DNA vector expressing PGRP-LCa^TM+Intra^. At 36 h post transfection, total RNA was isolated for quantitative RT-PCR to check transcriptional levels of *attacin* (**d**), *cecropin A1* (**e**) and *diptericin* (**f**). Error bars represent s.d. (*n*=3). For data from **b**–**f**, the two-tailed Student’s *t*-test was used to analyse statistical significance. NS, no significance, versus control groups.

Given that Pellino is involved in the *Drosophila* Toll-mediated innate immunity pathway, we then determined whether Pellino also regulated the IMD signalling pathway, and established another reporter system, Att-luciferase, in which the luciferase was driven by the *attacin* gene promoter, which responds to IMD immune signalling. As shown in Fig. 2a, overexpression of the active form of PGRP-LCa (PGRP-LCa^TM+Intra^)^30^,^31^ significantly induced *luciferase* expression in a dose-dependent manner. In contrast to the role of Pellino in regulating the Toll pathway, knockdown of *pellino* had no effect on luciferase activity induced by expression of PGRP-LCa^TM+Intra^ in S2 cells (Fig. 2b). Overexpression of pellino did not affect PGRP-LCa^TM+Intra^-induced Att-luciferase activity in transfected S2 cells (Fig. 2c). Thus, Pellino might be dispensable for *attacin* expression. To confirm this, we performed quantitative PCR assays to measure mRNA levels of *attacin* (*att*), *cecropin A1* (*cecA1*) and *diptericin* (*dpt*) genes, three downstream antimicrobial genes in the IMD pathway, in *pellino* knockdown and control S2 cells. We found that knockdown of *pellino* did not affect the induction of *att*, *cecA1* or *dpt* expression (Fig. 2d–f). A recent study has suggested that ecdysone could potentially induce expression of target genes of the IMD pathway by triggering PGRP-LC expression^32^. We next sought to test whether Pellino regulates the IMD signalling induced by ecdysone, and found that neither overexpression of Pellino nor knockdown of Pellino has apparent effect on the IMD signalling activity induced by the ecdysone treatment (Supplementary Fig. 3a–e). Collectively, our findings suggest that Pellino functions specifically in the *Drosophila* Toll-mediated, but not in the IMD-mediated innate immune pathway.

### Pellino regulates Toll-mediated immune responses *in vivo*

**Figure 3:**
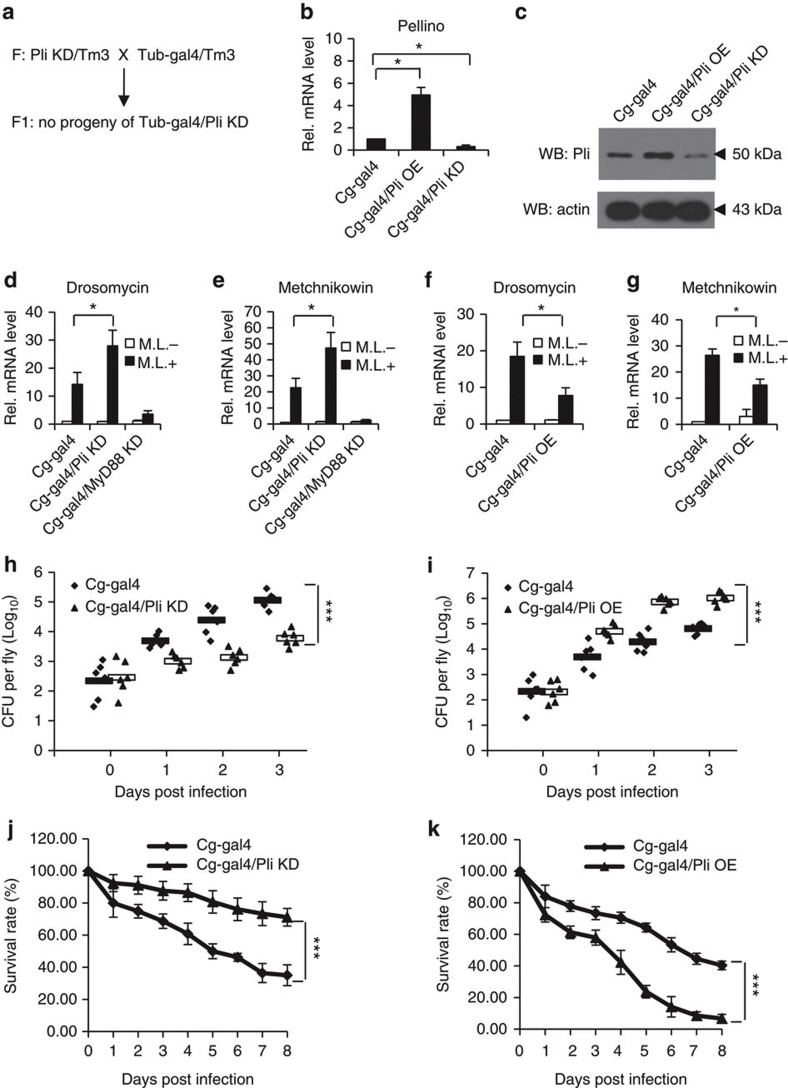
Pellino regulates Toll-mediated immune responses *in vivo*. (**a**) The UAS-GAL4 strategy was used to test the *in vivo* function of *pellino*. Ubiquitous knockdown of *pellino* by the *Tub-gal4* resulted in animal lethality at the late larval stage. (**b**,**c**) Flies containing overexpression or knockdown of *pellino* specifically in fat bodies by the Cg-gal4 driver were lysed to measure expression levels of *pellino* mRNA (**b**) or protein (**c**). Error bars represent s.d. (*n*=3). (**d**,**e**) *Pellino* knockdown, *MyD88* knockdown (positive control) and Cg-gal4 flies (wild-type control) were infected with *M. luteus* for 12 h and lysed for quantitative RT-PCR analysis to measure mRNA levels of *drosomycin* (**d**) and *metchnikowin* (**e**). Error bars represent s.d. (*n*=3). (**f**,**g**) *Pellino* overexpression flies and controls were infected with *M. luteus* for 12 h and then lysed for quantitative RT-PCR analysis to measure mRNA levels of *drosomycin* (**f**) and *metchnikowin* (**g**). Error bars represent s.d. (*n*=3). (**h**,**i**) *Pellino* knockdown (**h**) or overexpression (**i**) flies and controls were pricked with a needle previously dipped into *M. luteus*. Bacterial replication was monitored at different times as indicated. (**j**,**k**) *Pellino* knockdown (**j**) or overexpression (**k**) flies and controls were infected with *M. luteus* and monitored daily for mortality. Error bars represent s.d. (*n*=3). For data from **b** and **d**–**g**, the two-tailed Student’s *t*-test was used to analyse statistical significance. For data from **h** and **i**, the rANOVA test was used to analyse the variance of repeat measures. For data from **j** and **k**, the Log-rank test was used to analyse the variance of the survival rates between two groups. *P<0.05, ***P<0.001, versus control groups.

Next, we determined whether Pellino controlled the Toll-mediated immune pathway *in vivo*. As previously described^33^, we generated several transgenic lines, P{*UASp-artmiR-pellino*}, in which artificial miRNAs that specifically target the *pellino* gene were under the control of the *UASp* promoter. As shown in Fig. 3a and Supplementary Fig. 4a, ubiquitous expression of *artmiR-pellino* by the *Tub-gal4* driver efficiently downregulated Pellino expression, but caused animal lethality at the late larval stage, which was consistent with previous findings that *pellino* is an essential gene for animal development^25^. To assess the biological role of Pellino in regulating innate immunity in the adult stage of *Drosophila*, we first employed the ubiquitous Gal4–Gal80ts driver system and took advantage of the temperature-dependent activity of the Gal4 that is negatively regulated by the Gal80 to control expression of *artmiR-pellino* specifically at the adult stage. By using this system, crosses were performed and the resulting progeny were initially raised at the permissive temperature (18 °C) for the Gal80ts inhibitor to repress the Gal4 activity. Once adult eclosion, the newly progeny were then shifted to 29 °C to allow *artmiR-pellino* expression. The 6-day-old control and *pellino* knockdown flies were then infected by Gram-positive bacterium *Micrococus luteus* (*M. luteus*), a widely used bacterial strain that induces activation of the Toll-mediated immune response and expression of AMPs. As shown in Supplementary Fig. 4b,c, expression levels of *drosomycin* and *metchnikowin* mRNAs were higher in *pellino* knockdown flies than that in controls. Since the fat body is the *Drosophila* immune organ, we then employed the fat body-specific driver, *Cg-gal4*, to knock down *pellino* expression in fat bodies, and obtained consistent results (Fig. 3b–e). To further confirm these observations, we next used other bacterial and fungal pathogens, such as *Enterococcus faecalis* (*E. faecalis*) and *Aspergillus fumigatus* (*A. fumigatus*), to infect *wild-type* and *pellino* knockdown flies. As shown in Supplementary Fig. 4d–g, knockdown of *pellino* indeed enhanced Toll signalling activity induced by these pathogens. To test whether overexpression of pellino downregulates Toll signalling, we infected flies overexpressing *pellino* by the *Cg-gal4* driver with *M. luteus*, *E. faecalis* or *A. fumigatus* and found that elevation of Pellino expression in fat bodies significantly reduced the expression levels of *drosomycin* and *metchnikowin*, when compared with controls (Fig. 3f,g and Supplementary Fig. 4d–g). In agreement with the data from qRT-PCR assays, consistent results were obtained, when protein levels of Drosomycin and Defensin were measured (Supplementary Fig. 4h). Collectively, our results strongly argue that Pellino functions as a negative regulator to control Toll-mediated innate immunity in the *Drosophila* immune organ, the fat bodies.

### Pellino regulates invading pathogen growth

Given that Pellino has a negative role in controlling the Toll-mediated innate immunity pathway, we tested the biological impact of Pellino, and examined whether *pellino* influenced *M. luteus* growth in host flies by performing bacterial load assays as previously described^9^. Bacterial colony-forming unit (CFU) assays demonstrated the quantity of *M. luteus* colonies from *pellino* knockdown flies was lower than that from control flies (Fig. 3h), suggesting Pellino might influence the breeding of *M. luteus*. Comparison of the survival rate of control and *pellino* knockdown flies infected with *M. luteus* showed that *pellino* knockdown flies had a higher survival rate than controls (Fig. 3j). To confirm these observations, we tested whether fat body-specific overexpression of Pellino influenced the breeding of *M. luteus*. The quantity of *M. luteus* colonies from flies with fat body-specific overexpression of *pellino* was increased (Fig. 3i) compared with controls. In addition, the *pellino* overexpressed flies had a lower survival rate than controls (Fig. 3k). Thus, Pellino may influence the host defence ability against Gram-positive bacterial infection.

### Pellino physically interacts with MyD88

**Figure 4:**
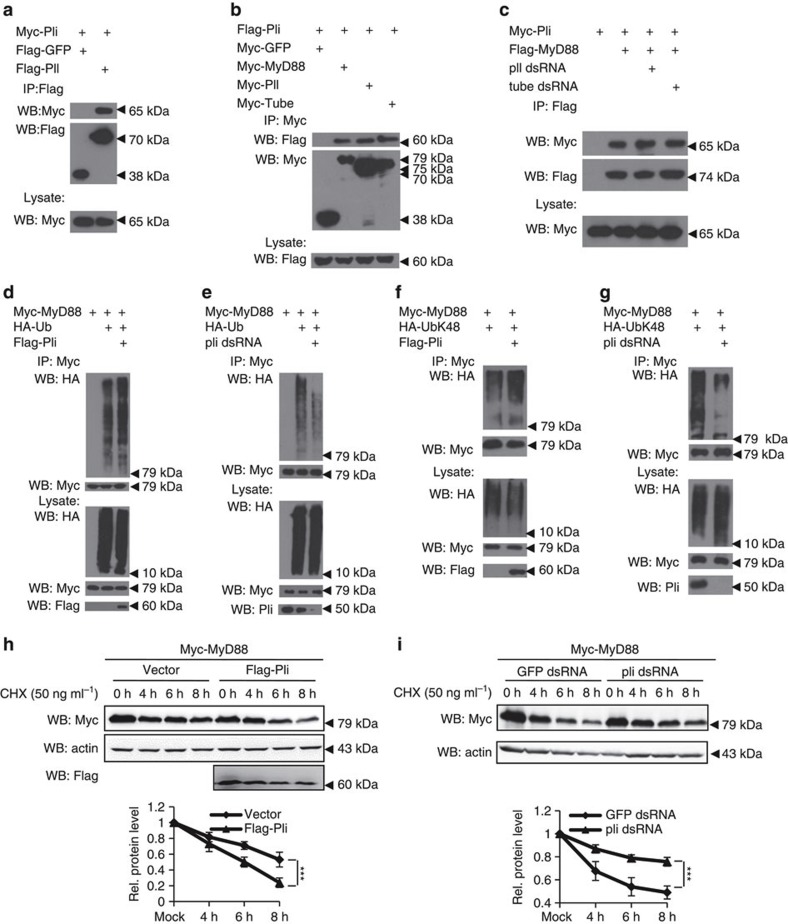
Pellino regulates MyD88 ubiquitination and turnover through K-48 linkage. (**a**,**b**) S2 cells were transfected with combinations of expression plasmids as indicated. Forty-eight hours after transfection, cell lysates were prepared, immunoprecipitated with anti-Flag beads (**a**) or anti-Myc beads (**b**), followed by immunoblot analysis with the indicated antibodies. Expression levels of transfected proteins in whole-cell lysates are shown in bottom panel. (**c**) S2 cells were treated with the dsRNAs as indicated for 48 h, and then transfected with combinations of expression plasmids as indicated. Forty-eight hours after transfection, cell lysates were prepared, immunoprecipitated with anti-Flag beads, followed by immunoblot analysis with the indicated antibodies. Expression levels of transfected proteins in whole-cell lysates are shown in bottom. (**d**,**e**) S2 cells were transfected with combinations of expression plasmids as indicated (**d**) or cells were also pretreated with *pellino* dsRNA (**e**). Cell lysates were then used to perform immunoprecipitation experiments with anti-Myc beads, followed by immunoblot analysis with anti-HA antibodies to show ubiquitination pattern of MyD88. (**f**,**g**) Similar to **d** and **e**, except the DNA vector expressing HA-Ub-K48 was used to examine the MyD88 K48-linked ubiquitination pattern under overexpression (**f**) or knockdown (**g**) of *pellino* condition. (**h**) S2 cells were transfected with expression constructs as indicated, and after 48 h post transfection, cells were treated with CHX (50 ng ml^−1^) for different times, followed by immunoblotting to check MyD88 expression levels. Densitometry analysis to quantify MyD88 expression is shown in the bottom panel. Error bars represent s.d. (*n*=3). (**i**) S2 cells were treated with dsRNAs targeting *gfp* or *pellino* for 48 h, then transfected with Myc-MyD88. After 48 h, cells were treated with CHX (50 ng ml^−1^) for different times, followed by immunoblotting to check MyD88 expression levels. Densitometry analysis to quantify MyD88 expression is shown in the bottom panel. Error bars represent s.d. (*n*=3). For data from **h** and **i**, the Log-rank test was used to analyse the variance of the protein stability between two groups. ***P<0.001, versus control groups.

To understand the molecular mechanism underlying the action of Pellino in the Toll-mediated innate immune pathway, we examined whether Pellino interacted with known components of the Toll pathway. Pellino has been previously reported to interact with Pelle in an yeast two-hybrid analysis^19^. Consistent with this, our co-immunoprecipitation assays showed that Pellino associated with Pelle in transfected S2 cells (Fig. 4a and Supplementary Fig. 5a). We then tested whether Pellino also associated with Tube and MyD88, other key components of the Toll signalling pathway^13^. Co-immunoprecipitation assays demonstrated that Tube and MyD88 were also associated with Pellino (Fig. 4b and Supplementary Fig. 5b,c). Previous studies showed that Tube and Pelle formed a functional complex with MyD88 to regulate Toll signalling. We therefore sought to test whether Pelle or Tube affects the MyD88–Pellino complex assembly. As shown in co-immunoprecipitation experiments, knockdown of *tube* or *pelle* (Supplementary Fig. 6) did not affect MyD88–Pellino interactions (Fig. 4c). Thus, Pellino forms a complex with MyD88 in a Pelle/Tube independent manner.

### Pellino regulates MyD88 ubiquitination and turnover

Given that Pellino functions as a ubiquitin E3 ligase and is physically associated with Pelle, Tube and MyD88, we tested whether Pellino controlled ubiquitination of these proteins by performing *in vivo* ubiquitination assays in transfected S2 cells. As shown in Fig. 4d, overexpression of Pellino markedly increased ubiquitination of MyD88, but did not ubiquitinate Pelle or Tube. Conversely, the conjugation of ubiquitin to MyD88 was markedly reduced when S2 cells were treated with dsRNAs of *pellino* (Fig. 4e), suggesting Pellino has a specific role in regulating ubiquitination of MyD88, rather than Pelle or Tube. It has been proposed that K48-linked ubiquitination is exclusively related to protein degradation, whereas K-63-linked ubiquitination likely promotes protein–protein association and signalling activation^34^. To determine whether Pellino-mediated MyD88 ubiquitination is linked via K63 or K48, we also employed a series of HA-tagged mutants of ubiquitin to perform ubiquitination assays. Overexpression or knockdown of Pellino markedly increased or reduced the ubiquitination of MyD88, respectively, when a HA-Ub-K48 mutant (all lysines in ubiquitin are mutated to arginine except K48) was used (Fig. 4f,g). In contrast, alteration of Pellino expression did not change the ubiquitination pattern of MyD88, when a HA-Ub-K63 mutant (all lysines in ubiquitin are mutated to arginine except K63) was used (Supplementary Fig. 7a,b). Thus, Pellino-mediated MyD88 ubiquitination occurs through K-48 ubiquitin linkage. In support of this, overexpression of Pellino reduced the expression levels of MyD88 (Fig. 4h), whereas knockdown of Pellino increased the stability of the Myc-tagged MyD88 and endogenous MyD88 as well (Fig. 4i and Supplementary Fig. 7c). Additionally, we also observed an apparent reduction of endogenous MyD88 upon high levels of Toll activation (Supplementary Fig. 7d). Collectively, our results strongly argue that Pellino interacts with MyD88 and specifically regulates MyD88 ubiquitination and turnover.

### Toll signalling promotes accumulation of Pellino at the plasma membrane

**Figure 5:**
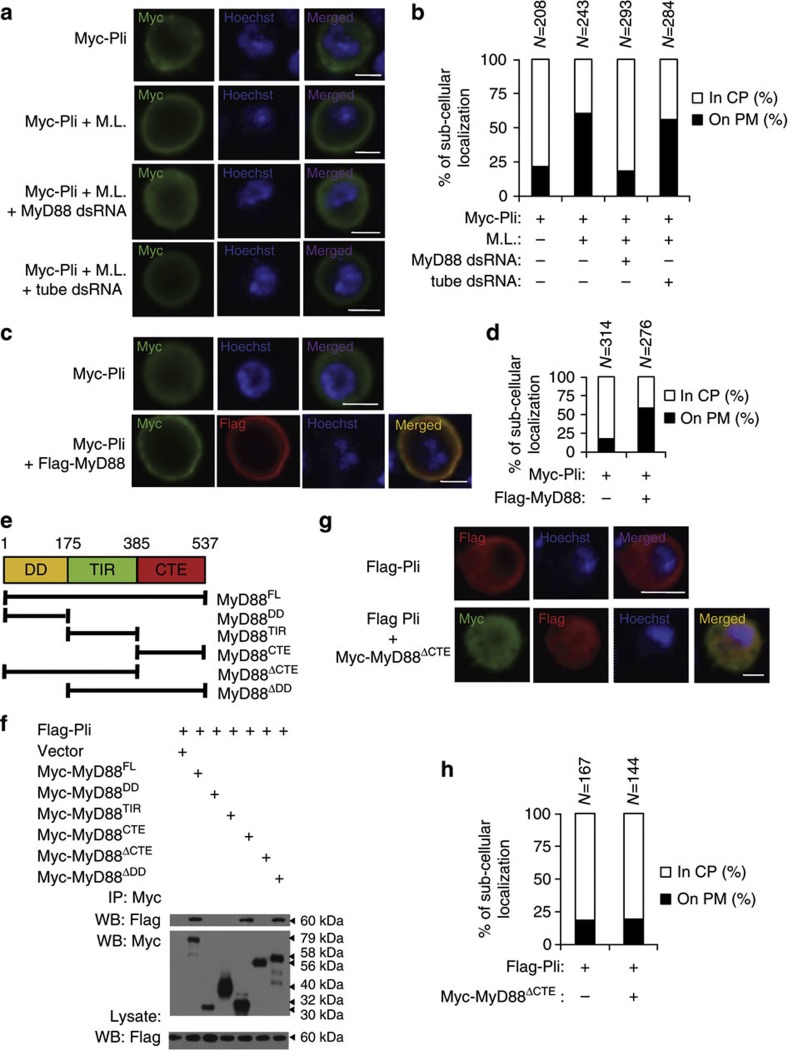
Toll signalling promotes Pellino to accumulate at the plasma membrane in a MyD88-dependent manner. (**a**) S2 cells were transfected with Myc-tagged Pellino or also pretreated with *MyD88 or Tube* dsRNA, and then treated with or without pre-boiled *M. luteus* (*ML*) for 12 h as indicated. Cells were further stained with anti-Myc antibody and Hoechst, and imaged by confocal microscopy. Scale bars, 10 μm. (**b**) Statistical assays of plasma membrane (PM) or cytoplasmic (CP) localization of Pellino in cell samples from (**a**). (**c**,**d**) S2 cells transfected with Myc-Pellino alone or Myc-Pellino in combination with Flag-MyD88 were stained with Hoechst and anti-Myc antibody alone or with anti-Flag antibody as indicated and imaged by confocal microscopy(**c**). Scale bars, 10 μm. (**d**) Statistical assays of PM or CP localization of Pellino in cell samples from (**c**). (**e**) Schematic diagram of MyD88 and its truncated mutants. (**f**) S2 cells were transfected with Flag-tagged Pellino and Myc-tagged MyD88 or its truncated mutants. Forty-eight hours after transfection, cell lysates were prepared, immunoprecipitated with anti-Myc beads, followed by immunoblot analysis with the indicated antibodies. (**g**,**h**) S2 cells transfected with Flag-Pellino alone or in combination with Myc-MyD88^ΔCTE^ and stained with Hoechst and anti-Flag antibody alone or with anti-Myc antibody as indicated and imaged by confocal microscopy (**g**). Scale bars, 10 μm. (**h**) Statistical assays of PM or CP localization of Pellino in cell samples from (**g**).

It has been shown that *Drosophila* MyD88 is localized at the plasma membrane in *Drosophila* embryos^18^. Recently, an elegant study demonstrated the plasma membrane localization of MyD88 is necessary and sufficient for the signalling transduction of Toll-mediated innate immune responses^17^. As Pellino negatively regulates Toll signalling by controlling the ubiquitination and degradation of MyD88, Pellino might function at the plasma membrane to target MyD88. To test this, we used S2 cells to perform cell-based immunostaining assays. Pellino was expressed predominately in the cytosolic region, but small portion of Pellino staining was observed at the plasma membrane when S2 cells expressed Myc-tagged Pellino alone (Fig. 5a,b). However, Pellino predominately accumulated at the plasma membrane, when cells were stimulated with pretreated *M. luteus* (seen in Methods) or activated by a gain-of-function mutant Toll^10B^,^35^,^36^ (Fig. 5a,b and Supplementary Fig. 8a,b), suggesting that activation of Toll signalling is sufficient to promote the plasma membrane accumulation of Pellino. To test whether MyD88 or Tube is required for Pellino plasma membrane accumulation, we performed *MyD88* or *Tube* knockdown experiments in S2 cells, inactivation of MyD88 but not Tube markedly reduced *M. luteus*- or Toll^10B^-induced Pellino plasma membrane accumulation in S2 cells (Fig. 5a,b and Supplementary Fig. 8a,b). Thus, the activation of Toll signalling promoted the accumulation of Pellino at the plasma membrane in a MyD88-dependent manner.

### MyD88 CTE domain is key for MyD88–Pellino regulatory loop

**Figure 6:**
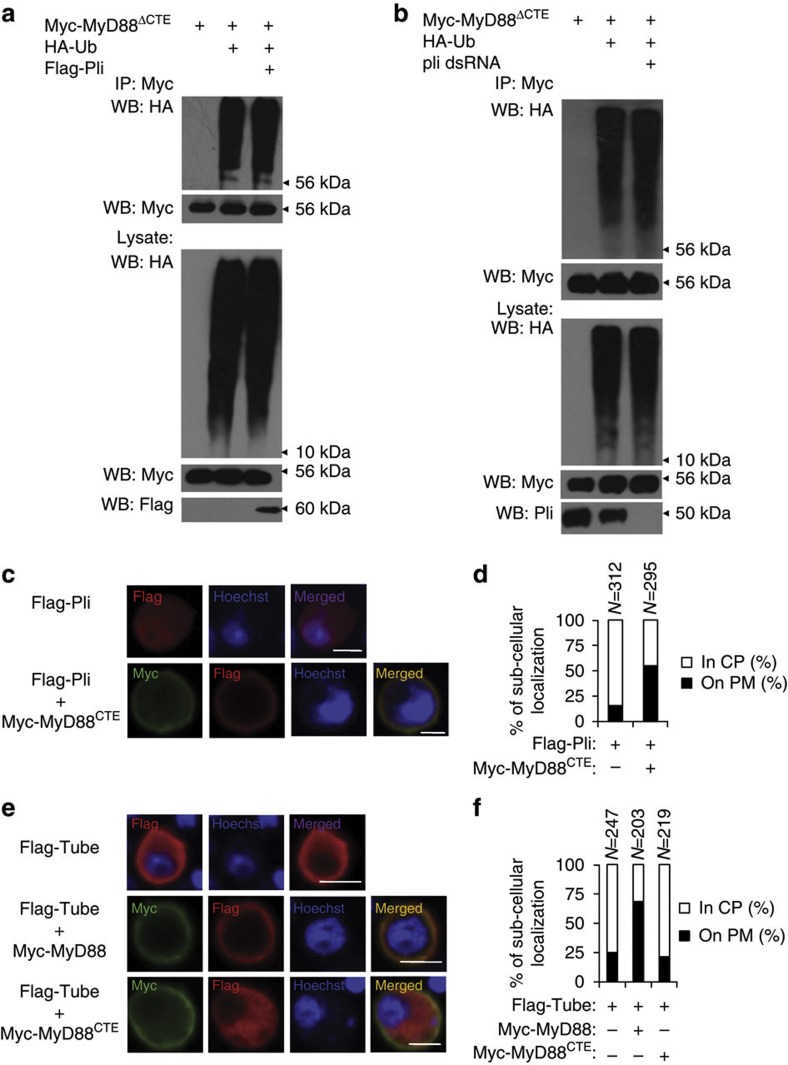
Pellino-mediated MyD88 ubiquitination is dependent on CTE domain. (**a**,**b**) S2 cells were transfected with Myc-MyD88^ΔCTE^ and HA-Ub with Flag-Pellino (**a**), or also pretreated with *pellino* dsRNA (**b**). Cell lysates were used to perform immunoprecipitation experiments with anti-Myc beads followed by immunoblot analysis with anti-HA antibodies to show ubiquitination patterns of MyD88^ΔCTE^. Expression levels of transfected proteins and Pellino knockdown efficiency in whole-cell lysates are shown in the bottom panel. (**c**,**d**) S2 cells transfected with Flag-Pellino alone or in combination with Myc-tagged MyD88 CTE domain were stained with Hoechst and anti-Flag antibody alone or with anti-Myc antibody as indicated and imaged by confocal microscopy (**c**). Scale bars, 10 μm. (**d**) Statistical assays of PM or CP localization of Pellino in transfected cells in panel (**c**). (**e**,**f**) S2 cells transfected with Flag-Tube alone or in combination with Myc-tagged MyD88 or MyD88 CTE domain were stained with Hoechst and anti-Flag antibody alone or with anti-Myc antibody as indicated and imaged by confocal microscopy (**e**). Scale bars, 10 μm. (**f**) Quantification assays of P.M. or C.P. localization of Tube in transfected cells in (**e**).

We next tested whether MyD88 was sufficient to induce the plasma membrane accumulation of Pellino. Plasma membrane accumulation of Pellino significantly increased when S2 cells were co-expressed with Myc-Pellino and Flag-MyD88, compared with Pellino expressed alone in S2 cells (Fig. 5c,d). To investigate the molecular basis of how MyD88 promotes plasma membrane accumulation of Pellino, we identified the specific domain in MyD88 required for Pellino interactions and generated a series of mutant forms of MyD88 constructs (Fig. 5e). Co-immunoprecipitation demonstrated that although DD and TIR domains were not required for MyD88 interactions with Pellino, the MyD88 CTE domain (a phosphoinositide-binding domain at the C-terminal of MyD88)^17^ was essential since the mutant form of MyD88^ΔCTE^ lacking CTE domain did not associate with Pellino (Fig. 5f). To test whether the CTE domain was required for MyD88 to promote the plasma membrane accumulation of Pellino, we co-overexpressed the mutant form of MyD88^ΔCTE^ and Pellino in S2, and found that, unlike wild-type MyD88, MyD88^ΔCTE^ failed to promote Pellino plasma membrane accumulation (Fig. 5c,d,g and h). Thus, the CTE domain is required for MyD88 plasma membrane localization, MyD88–Pellino interaction and MyD88 recruitment of Pellino plasma membrane accumulation. We next tested whether the MyD88 CTE domain was involved in Pellino-mediated MyD88 ubiquitination and degradation. Either overexpression or knockdown of Pellino did not affect the ubiquitination status and stability of MyD88^ΔCTE^, suggesting that Pellino regulates MyD88 ubiquitination through the CTE domain (Fig. 6a,b and Supplementary Fig. 7e). Given that the specific interaction of Pellino with the MyD88 CTE domain, we determined whether the MyD88 CTE domain was sufficient to promote plasma membrane localization by co-expression of Pellino and the MyD88 CTE domain in S2 cell cultures. Compared with the expression of Pellino alone, co-expression of Pellino with the MyD88 CTE domain resulted in significant cell-surface accumulation of Pellino (Fig. 6c,b). To determine the specificity of the CTE domain of MyD88 for the sub-cellular localization of Pellino, we tested whether MyD88 CTE was sufficient to recruit Tube onto the cell surface. Overexpression of full-length MyD88 resulted in the cell-surface accumulation of Tube, but MyD88 CTE alone did not (Fig. 6e,f). Taken together, our findings establish a model by which the activation of MyD88 promotes Pellino to accumulate onto the plasma membrane, where Pellino targets MyD88 for ubiquitination and degradation. This reciprocal regulation between MyD88 and Pellino appears to be mediated by the CTE domain of MyD88.

### Role of plasma membrane localization of MyD88 and Pellino

**Figure 7:**
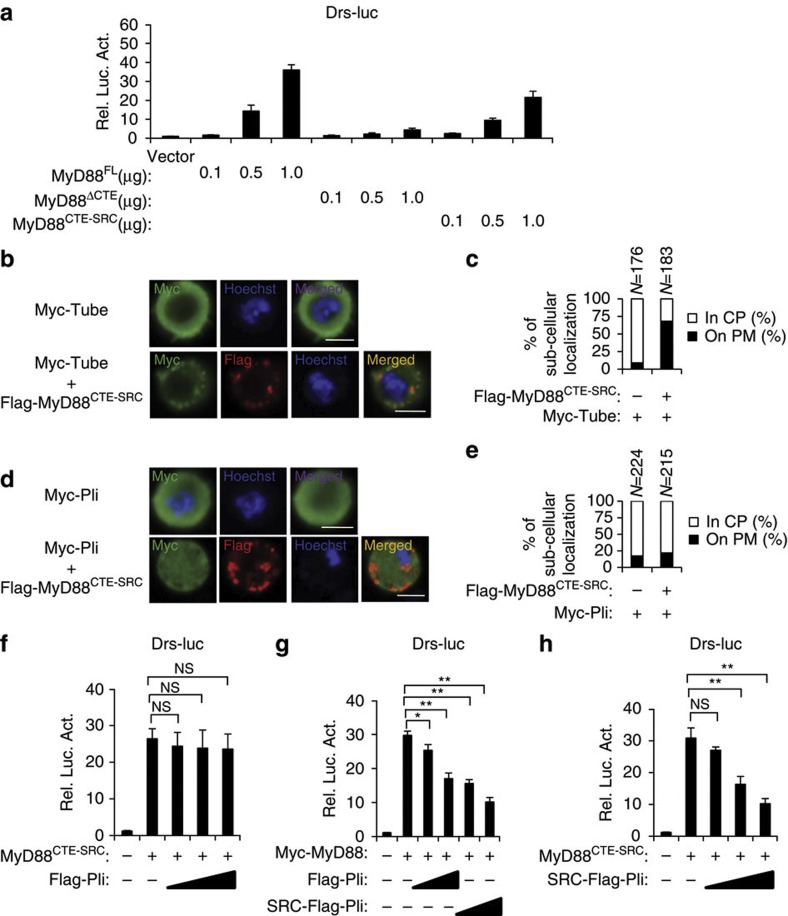
Cell-surface localization of MyD88 and Pellino balances Toll-mediated immune signalling. (**a**) S2 cells were transiently transfected with increasing amounts of DNA vectors expressing wild-type MyD88, MyD88^ΔCTE^ or MyD88^CTE–SRC^ with Drs-luc and Renilla-luc plasmids. Thirty-six hours after transfection, luciferase activity was measured and normalized based on Renilla luciferase activity. Results are presented relative to the luciferase activity in control cells. Error bars represent s.d. (*n*=3). (**b**,**c**) S2 cells transfected with Myc-Tube alone or in combination with Flag-tagged MyD88^CTE–SRC^ were stained with Hoechst and anti-Myc antibody alone or with anti-Flag antibody as indicated and imaged by confocal microscopy (**b**). Scale bars, 10 μm. (**c**) Quantification assays of P.M. or C.P. localization of Tube in transfected cells from (**b**). (**d**,**e**) Similar to (**b**,**c**), except expressing Myc-Pellino vector was used. Scale bars, 10 μm. (**f**) Luciferase reporter assays showing the effects of increasing expression of Pellino on luciferase activity induced by expression of MyD88^CTE–SRC^. S2 cells were transfected with vector or expression constructs as indicated. Thirty-six hours after transfection, cells were extracted for reporter assay. Error bars represent s.d. (*n*=3). (**g**,**h**) Luciferase reporter assays showing the effects of increasing expression of wild-type Pellino or the SRC-tagged Pellino on luciferase activity induced by expression of wild-type MyD88 (**g**) or SRC-Pellino on the luciferase activity induced by expression of MyD88^CTE–SRC^ (**h**). S2 cells were transfected with vector or expression constructs as indicated. Thirty-six hours after transfection, cells were lysed for reporter assay. Error bars represent s.d. (*n*=3). For data from **f**–**h**, the two-tailed Student’s *t*-test was used to analyse statistical significance. *P<0.05, **P<0.01, NS, no significance, versus control groups.

Given that the CTE domain is essential for MyD88 plasma membrane localization and its function in Toll-mediated immune signalling transduction, we tested whether plasma membrane localization was sufficient for MyD88 function. We generated a plasma membrane-localized mutant form of MyD88, in which the SRC domain replaced the CTE domain (referred as MyD88^CTE–SRC^)^37^. We noted that, although a portion of protein exhibited particulate distribution, majority of MyD88^CTE–SRC^ localized to plasma membrane (Fig. 7b,d).We then performed a luciferase reporter assay, as shown in Fig. 7a, while expression of MyD88^ΔCTE^ failed to stimulate luciferase reporter expression, the replaced mutant form, MyD88^CTE–SRC^, was efficient to induce Toll signalling, mimicking wild-type MyD88. This suggested that plasma membrane localization is essential for the MyD88 induction of Toll signalling. In support of this, expression of the SRC-replaced form of MyD88^CTE–SRC^ was sufficient to recruit the Tube protein onto the plasma membrane, given the TIR domain of Tube was required for interactions between Tube and MyD88 (Fig. 7b,c). However, the expression of the SRC-replaced form of MyD88^CTE–SRC^ was not sufficient to recruit Pellino to accumulate at the plasma membrane (Fig. 7d,e), emphasizing the CTE domain was specifically required for MyD88-induced Pellino plasma membrane localization. We next tested whether overexpression of wild-type Pellino affected MyD88^CTE–SRC^-induced Toll signalling. Overexpression of wild-type Pellino had no effect on Toll signalling induced by MyD88^CTE–SRC^ (Fig. 7f). As the expression of membrane-localized Pellino, SRC-Pellino, enhanced its inhibitory activity on Toll signalling induced by the wild-type form of MyD88 (Fig. 7g), we then tested whether SRC-Pellino efficiently inhibited the function of MyD88^CTE–SRC^. SRC-Pellino inhibited Toll signalling activity induced by MyD88^CTE–SRC^ in a dose-dependent manner (Fig. 7h). Thus, our findings strongly argue that the plasma membrane localization of MyD88 and Pellino balances Toll-mediated immune signalling.

## Discussion

In *Drosophila*, the Toll signalling is essential for embryonic development and innate immune responses^3^,^4^. However, how Toll signalling is regulated to maintain homeostasis of host innate immunity remains poorly understood. In this study, we demonstrated that *Drosophila* Pellino, a RING-containing E3 ligase, functions as a negative regulator in the Toll-mediated innate immunity pathway by targeting MyD88. Importantly, we observed that MyD88 was associated with Pellino and promoted Pellino accumulation onto the plasma membrane, where Pellino targeted MyD88 for ubiquitination and degradation. We thus uncovered a novel feedback regulatory loop involving MyD88 and Pellino that balanced Toll-mediated signalling, thereby maintaining homeostasis of host innate immunity.

Ubiquitin-mediated protein modification and/or degradation has a variety of roles in the regulation of many cellular and developmental processes^38^. The enzymatic reaction of protein ubiquitination is a coordinated three-step process involving three classes of enzymes, E1 (ubiquitin-activating enzyme), E2 (ubiquitin-conjugating enzyme) and E3 (ubiquitin ligase)^38^. Upon pathway activation, the MyD88–Tube–Pelle complex promotes the phosphorylation and degradation of Cactus, the *Drosophila* IκB factor, by Slimb/βTrCP by a ubiquitin-dependent mechanism^18^,^39^. In addition, biochemical studies have revealed a mechanism through which the K-63 linkage of ubiquitin promotes the TRAF6/dTRAF2-mediated activation of IKK and NF-κB^34^,^40^. These findings highlight that ubiquitination plays important roles in the regulation of Toll signalling. Members of the Pellino family are RING-containing proteins and were previously shown to function as ubiquitin E3 ligases to regulate TLR-mediated innate immune responses either positively or negatively in mammals^20^,^21^. Although the Drosophila genome has only one member, a previous study proposed that *Drosophila* Pellino acted as a positive regulator in Toll signalling, since mutation of *pellino* resulted in impaired *Drosomycin* expression and reduced survival against Gram-positive bacterial infection^25^. In this study, we used a cell-based luciferase-reporter assay system and performed an RNAi knockdown screen. From this screen, we were surprised to find that while knockdown of *pellino* increased activation of the *Drosomycin* promoter induced by overexpression of active Toll receptor, overexpression of Pellino suppressed the activity of Toll signalling in a dose-dependent manner in luciferase reporter assays. These findings suggest that *Drosophila* Pellino has a negative role in the regulation of Toll signalling in S2 cells. We further assessed the *in vivo* function of Pellino by generating knockdown and overexpression of Pellino transgenes. Notably, tissue-specific knockdown or overexpression of *pellino* in the fat body supported the findings that Pellino negatively regulates Toll signalling activity *in vivo*. In addition, alteration of *pellino* in the fat body influenced *M. luteus* growth in host flies, further emphasizing the biological impact of *pellino* in negatively regulating the innate immune response. Collectively, our findings strongly support the idea that *Drosophila* Pellino plays a negative rather than positive role in the Toll signalling pathway.

*Drosophila* Pellino was initially identified as a Pelle-interacting protein^19^. However, the molecular basis of the action of *Drosophila* Pellino in Toll signalling remains unknown. In this study, we demonstrated that Pellino not only forms a complex with Pelle but also associates with both Tube and MyD88. Thus, Pelle, Tube and/or MyD88 could be the downstream target(s) of Pellino, given that Pellino functions as a ubiquitin E3 ligase^20^,^21^. Our biochemical assays showed that alteration of Pellino by knockdown or overexpression markedly changed the ubiquitination pattern and stability status of MyD88, upon pathway activation, indicating MyD88 is the primary target of Pellino in the Toll signalling pathway. A recent study showed MyD88 functions as an adaptor of the Toll receptor and is mainly present at the plasma membrane to activate the downstream events of Toll/MyD88 (ref. 17). Interestingly, we demonstrated that upon bacterium stimulation, high levels of Pellino accumulated onto the plasma membrane, which was dependent on MyD88 activity. Thus, the plasma membrane localization of Pellino might be important for its targeting of MyD88, which then downregulates Toll signalling activity. In support of this, we found that expression of the plasma membrane-tagged Pellino form, SRC-Pellino, strongly enhanced its activity in downregulation of Toll signalling, compared with its wild-type form. Given that MyD88 is associated with Pellino, we have identified a novel feedback mechanism by which MyD88 recruits Pellino onto the plasma membrane, where Pellino targets MyD88 for ubiquitination and degradation.

In mammals, it has been shown that the function of Pellino proteins is regulated by the IRAK-1, the homologue of Pelle, through phosphorylation in a site-specific manner. Interestingly, we found that some of these sites are also present in *Drosophila* Pellino. By generating a series of mutant forms of Pellino, in which the S or T was individually or in combination mutated to A, we found that these mutations did not affect the function of Pellino, as indicated by luciferase reporter assays (Supplementary Fig. 9a), as well as the interaction of Pellino with MyD88 in co-immunoprecipitation assays (Supplementary Fig. 9b). Moreover, Pellino carrying these mutations could accumulate at plasma membrane upon Toll signalling activation (Supplementary Fig. 9c,d). Collectively, the conserved sites are not required for E3 ligase activity of *Drosophila* Pellino in the regulation of MyD88.

Pathways involving signalling transduction are most often initiated by transmembrane receptors, which typically possess separate domains for engaging extracellular ligands and intracellular adaptor proteins^15^. In the case of Toll signalling pathway, the plasma membrane localization of MyD88 is necessary for Toll signalling transduction by recruiting the downstream adaptor protein Tube onto the plasma membrane^17^,^18^. *Drosophila* MyD88 contains a phosphoinositide-binding domain (named CTE) at its C terminus, which is proposed to function as a ‘SORT’ adaptor for MyD88 and Tube cell-surface localization, and subsequently for downstream signalling transduction of the Toll pathway^17^. The CTE domain of MyD88 is the functional homologue of TIRAP in mammals^17^,^41^,^42^, and thus positively regulates Toll signalling. In this study, in addition to its positive role in Toll signalling, MyD88 CTE was sufficient to interact with and promoted Pellino cell-surface localization. The plasma membrane localization of Pellino allows the negative control of MyD88 ubiquitination and degradation in a feedback regulatory manner. Thus, the dual roles of the MyD88 CTE domain are essential for both the activation and negative maintenance of homeostasis of Toll signalling.

The endocytosis is a dynamic cellular process controlling various signalling pathways in either a positive or a negative manner. Recently, several studies have shown that the endocytic pathway is involved in regulating the Toll signalling transduction in Drosophila^43^,^44^. It has been shown that the endosomal proteins, such as Mop and Hrs, colocalize with the Toll receptor in endosomes and function genetically upstream of MyD88 and Pelle; thus, endocytosis is required for the Toll signalling activation. Internalization and trafficking of the nascent receptor to some particular endosomes has been proposed to extend signalling activity, as in the cases of Notch and JAK/STAT signalling pathways in *Drosophila*^45^,^46^. In support of this, we found that expression of the hyper-activated form of MyD88, MyD88^CTE–SRC^, not only showed a plasma membrane localization but also exhibited a particulate distribution pattern in cytosolic region. Notably, we observed that addition of pretreated *M. luteus* into S2 cells could promote the Pellino plasma membrane localization (Fig. 5a) and activate Toll signalling, albeit at a relative lower level, when compared with the control cells stimulated by activated Toll receptor. Knockdown of *Croquemort* (*crq*), a component of phagocytosis pathway^47^, significantly suppressed Pellino plasma membrane localization and upregulation of *metchnikowin*, *defensin* and *drosomycin* induced by *M. luteus* in S2 cells (Supplementary Fig. 10a–h). These findings together emphasize the significant roles of some specific type of endocytic (or phagocytic) vesicle-mediated Toll signalling pathway. It would be interesting to test how the endocytic (or phagocytic) pathway contributes to the regulation of *Drosophila* innate immunity in the future studies.

## Methods

### *Drosophila* strains

Fly stocks were maintained under standard culture conditions. The *w*^*1118*^ strain was used as a control and the host for P-element-mediated transformation^48^. The following strains were used in this study: (1) P{*UASp-pellino*}, in which the full-length pellino was placed under the control of the UASp promoter^49^; (2) *Pellino* knockdown transgenes, P{*UASp-ArtmiR-pellino*} that express artificial microRNAs targeting *pellino* mRNA were generated by the following method. Briefly, the two pairs of designed primers were denatured and annealed to form short dsDNA, which were then digested by NheI and EcoRI enzymes to insert into UASp KN and BX vectors, respectively. The two inserted UASp vectors were then digested with BamHI and XbaI enzymes, producing a short DNA and a broken vector. The final UASp–ArtmiR–pellino vector was obtained when the short DNA from digested UASp KN vector was inserted into the broken UASp BX vector; (3) the Cg-gal4, a fat-body-specific gal4 driver, was a kind gift from Dr Sheng Li (Shanghai Institutes for Biological Science, CAS); (4) P{*tubP-gal4*} was from the Bloomington Stock Center (BL5138). (5) The ubiquitous Gal4–Gal80ts driver system was used to express the target genes specifically at the adult stage. Crosses were performed and the resulting progeny were initially raised at the permissive temperature (18 °C) for the Gal80ts inhibitor to repress the Gal4 activity. Once adult eclosion, the newly progeny were then shifted to 29 °C to allow activation of Gal4 at the adult stage.

Primers used for transgene vector construction are shown in Supplementary Table 1.

### Immunoprecipitation and western blot assay

S2 cells were cultured in insect medium (Hyclone) at 27 °C. DNA transfection was performed using Lipofectamine 2000 (Invitrogen) or using the standard calcium phosphate transfection method. For immunoprecipitation assays, cells were lysed in lysis buffer (150 mM NaCl, 50 mM Tris-HCl, pH 7.4, 10% glycerol, 0.5% Triton X-100, 10 μg ml^−1^ aprotinin, 10 μg ml^−1^ leupeptin and 1 mM phenylmethylsulfonyl fluoride). Anti-Flag M2 affinity gel (Sigma) or anti-Myc affinity gel (Abmart) were used for indicated immunoprecipitation experiments. The immune complexes were washed three times with lysis buffer containing 150 mM NaCl and subjected to immunoblot analysis with the indicated antibodies. Immunoblotting was performed according to standard procedures. The following antibodies were used for western blotting: rabbit anti-Myc (1:3,000, MBL), rabbit anti-HA (1:3,000, MBL), rabbit or mouse anti-Flag (1:2,000, Sigma) and rabbit anti-Actin (1:2,000, Sigma). The antibody against Pellino was generated by immunizing rabbits with the recombinant proteins His_6_-Pellino (1–120) produced in *E. coli.* The antibodies against MyD88, Drosomycin and Defensin were generated by immunizing mice with the *E*. *coli*-produced recombinant proteins His_6_-MyD88 (63–163), Drosomycin (full-length) and Defensin (full-length) respectively. Original western blots can be found in Supplementary Fig. 11.

### *In vivo* ubiquitination assay

*In vivo* ubiquitination assays were performed by the following method. Briefly, S2 cells were transfected with the indicated DNA constructs. At 48 h post-transfection, cells were treated with MG132 (final concentration 50 μM) and/or NH_4_Cl (final concentration 50 mM) for 6 h. Cells were lysed in lysis buffer (150 mM NaCl, 50 mM Tris-HCl, pH 7.4, 10% glycerol, 1% Triton X-100, 0.1% SDS and 10 mM NEM). After pull-down with anti-Myc affinity beads for 4 h, the beads were extensively washed three times with lysis buffer containing 0.1% SDS and 500 mM NaCl for a total of 1 h. Samples were then subjected to western blot analysis.

### Toll and IMD signalling reporter assay in S2 cells

Toll and IMD signalling reporter assays in S2 cells were performed using the *drosomycin–luciferase* or *attacin*–*luciferase* constructs, in which the luciferase coding sequence was placed under the *drosomycin* or *attacin* promoter. For normalizing the efficiency of the transfection, the *actinP–Renilla* construct was used. Firefly luciferase and Renilla luciferase were performed by the following method. In brief, the S2 cells were lysed with 50 μl of Passive lysis buffer (Promega) for luciferase assays using Luciferase assay reagent (Promega), according to the manufacturer's instructions.

### Toll signalling induced by pre-boiled *M. luteus*

*M. luteus* was cultured overnight in 30 °C and harvested when the OD_600_ of the culture material reached 1.00. The bacteria collection was then diluted 10 times with PBS buffer and boiled for 20 minutes. S2 cells were treated with this pre-boiled bacterial mixture for 12 h and then harvested for quantitative RT-PCR or immunostaining assays.

### RNAi knockdown assays in S2 cells

All dsRNAs were synthesized according to the standard protocol. S2 cells were collected and diluted into fresh medium at a density of 1.0 × 10^6^ cells per ml, and then were treated with dsRNA immediately. Primers for dsRNA synthesis are shown in Supplementary Table 2.

### Quantitative RT-PCR

Total RNA was isolated with Trizol Reagent (Invitrogen) according to the manufacturer’s instructions and cDNA was synthesized using SuperScript III First-Strand cDNA Synthesis kit (Invitrogen). Quantitative RT-PCR was performed using SYBR Premix Ex Taq (Takara) in triplicate on a Bio-Rad iCycler iQ5 PCR Thermal Cycler. Template concentrations were normalized to endogenous reference rp49. Primers for quantitative RT-PCR are shown in Supplementary Table 3.

### Strains of bacteria

All the bacterium strains used in this work were obtained from the China General Microbiological Culture Collection Center (CGMCC). The CGMCC numbers for *Micrococcus luteus*, *Enterococcus faecalis* and *Aspergillus fumigatus* are 1.2299, 1.2135 and 3.08027, respectively.

### Survival and bacterial CFU assays in flies

Bacterial challenge was performed by the following method. Briefly, a thin needle dipped into a concentrated overnight culture of *M. luteus* was used to prick 6-day-old adult flies for bacterial infection. After infection, flies were immediately transferred to fresh vials every day. Survival experiments were performed in the same conditions for each tested genotype, and 30 flies per group were tested for each genotype. The numbers of surviving flies were counted every day. Flies that died within 3 h (<5% of the total) after challenge were not considered in the analysis.

For CFU counting assays, 10 infected flies were harvested at the indicated times after infection and crushed in PBS buffer. The mixture was diluted serially, and 100 μl of each dilution was spread onto LB agar plates. Yellow bacterial clones were counted after overnight growth in a 30 °C culture hood.

### Cell immunofluorescence

For cell immunostaining experiments, cells were transferred onto a poly-lysine-treated cover glass 12 h after transfection. Twenty-four hours later, cells were fixed with 4% formaldehyde in PBS containing 0.1% Tween-20 for 15 min, permeabilized and blocked with 0.3% Tween-20 in PBS containing 5% BSA for 15 min at room temperature. Cells were then incubated with primary antibody and secondary antibody. Imaging of the cells was performed using a Zeiss LSM 710 META laser scanning confocal system. The following antibodies were used in this study: mouse anti-Flag (1:2,000, Sigma) and rabbit anti-Myc (1:2,000, MBL). The following secondary antibodies were used at a 1:3,000 dilution: goat anti-mouse Alexa555 and goat anti-rabbit Alexa488.

### Two-tailed Student’s *t*-test

The data of reporter and quantitative RT-PCR assays in this study were collected from three biological replicates. All statistical analyses are shown as means ±s.d. Significant differences between values under different experimental conditions were determined by two-tailed Student’s *t*-test. For all tests, a *P*-value <0.05 was considered statistically significant. *P<0.05, **P<0.01, ***P<0.001, NS, no significance, versus control groups.

### Log-rank test

The data of survival and protein stability assays were collected from three biological replicates. All statistical analyses are shown as means ±s.d. Significant differences were analysed by Log-rank test using the PASW Statistics 18 software. For all tests, a *P*-value <0.05 was considered statistically significant.

### Repeated measures analysis of variance for bacterial loading

The data of bacterial loading assays were collected from six biological replicates. The rANOVA, a commonly used statistical approach for repeated-measure designs, was used to analyse the variance of the bacterial loading assays. With such designs, the repeated-measure factor (the qualitative independent variable) is the with-subjects factor, while the dependent quantitative variable on which each participant is measured is the dependent variable. All statistical analyses results are shown as *xy* scatter diagrams. The variance analyses of these repeated measures were finished using the PASW Statistics 18 software. The *P*-value showed the results of the between-subjects effect tests.

### Flybase numbers for genes used in this study

The mRNA sequence data for genes of *Drosophila* described in this study can be found in the Flybase under the following accession numbers: Toll (NP_524518), Myd88 (NP_610479), Pelle (NP_476971), Tube (NP_001189164), Pellino (NP_524466) and PGRP-LCa (NP_729468).

## Additional information

**How to cite this article:** Ji, S. *et al.* Cell-surface localization of Pellino antagonizes Toll-mediated innate immune signalling by controlling MyD88 turnover in *Drosophila*. *Nat. Commun.* 5:3458 doi: 10.1038/ncomms4458 (2014).

## Author contributions

S.J., D.C. and Q.S. conceived and designed the experiments. S.J., M.S., L.L. and L.S. performed the experiments. S.J., M.S., D.C. and Q.S. analysed the data. X.Z., S.J performed all statistical analysis. S.J., D.C. and Q.S. wrote the manuscript.

## Supplementary Material

Supplementary Information Supplementary Figures 1-11 and Supplementary Tables 1-3

## References

[b1] Akira S., Uematsu S. & Takeuchi O. Pathogen recognition and innate immunity. Cell 124, 783–801 (2006). 16497588 10.1016/j.cell.2006.02.015

[b2] Alarco A. M. *et al.* Immune-deficient Drosophila melanogaster: a model for the innate immune response to human fungal pathogens. J. Immunol. 172, 5622–5628 (2004). 15100306 10.4049/jimmunol.172.9.5622

[b3] Hultmark D. Drosophila immunity: paths and patterns. Curr. Opin. Immunol. 15, 12–19 (2003). 12495727 10.1016/s0952-7915(02)00005-5

[b4] Hoffmann J. A. The immune response of Drosophila. Nature 426, 33–38 (2003). 14603309 10.1038/nature02021

[b5] Tanji T., Hu X., Weber A. N. & Ip Y. T. Toll and IMD pathways synergistically activate an innate immune response in Drosophila melanogaster. Mol. Cell Biol. 27, 4578–4588 (2007). 17438142 10.1128/MCB.01814-06 PMC1900069

[b6] Tanji T. & Ip Y. T. Regulators of the Toll and Imd pathways in the Drosophila innate immune response. Trends Immunol. 26, 193–198 (2005). 15797509 10.1016/j.it.2005.02.006

[b7] Hedengren M. *et al.* Relish, a central factor in the control of humoral but not cellular immunity in Drosophila. Mol. Cell 4, 827–837 (1999). 10619029 10.1016/s1097-2765(00)80392-5

[b8] Han Z. S. & Ip Y. T. Interaction and specificity of Rel-related proteins in regulating Drosophila immunity gene expression. J. Biol. Chem. 274, 21355–21361 (1999). 10409696 10.1074/jbc.274.30.21355

[b9] Lemaitre B., Nicolas E., Michaut L., Reichhart J. M. & Hoffmann J. A. The dorsoventral regulatory gene cassette spatzle/Toll/cactus controls the potent antifungal response in Drosophila adults. Cell 86, 973–983 (1996). 8808632 10.1016/s0092-8674(00)80172-5

[b10] Meng X., Khanuja B. S. & Ip Y. T. Toll receptor-mediated Drosophila immune response requires Dif, an NF-kappaB factor. Genes Dev. 13, 792–797 (1999). 10197979 10.1101/gad.13.7.792 PMC316597

[b11] Ligoxygakis P., Pelte N., Hoffmann J. A. & Reichhart J. M. Activation of Drosophila Toll during fungal infection by a blood serine protease. Science 297, 114–116 (2002). 12098703 10.1126/science.1072391

[b12] Horng T. & Medzhitov R. Drosophila MyD88 is an adapter in the Toll signaling pathway. Proc. Natl. Acad. Sci. USA 98, 12654–12658 (2001). 11606776 10.1073/pnas.231471798 PMC60109

[b13] Tauszig-Delamasure S., Bilak H., Capovilla M., Hoffmann J. A. & Imler J. L. Drosophila MyD88 is required for the response to fungal and Gram-positive bacterial infections. Nat. Immunol. 3, 91–97 (2002). 11743586 10.1038/ni747

[b14] Rutschmann S. *et al.* The Rel protein DIF mediates the antifungal but not the antibacterial host defense in Drosophila. Immunity 12, 569–580 (2000). 10843389 10.1016/s1074-7613(00)80208-3

[b15] Haugh J. M. A unified model for signal transduction reactions in cellular membranes. Biophys. J. 82, 591–604 (2002). 11806904 10.1016/S0006-3495(02)75424-6 PMC1301871

[b16] Kholodenko B. N., Hoek J. B. & Westerhoff H. V. Why cytoplasmic signalling proteins should be recruited to cell membranes. Trends Cell Biol. 10, 173–178 (2000). 10754559 10.1016/s0962-8924(00)01741-4

[b17] Marek L. R. & Kagan J. C. Phosphoinositide binding by the Toll adaptor dMyD88 controls antibacterial responses in Drosophila. Immunity 36, 612–622 (2012). 22464168 10.1016/j.immuni.2012.01.019 PMC3354765

[b18] Sun H., Towb P., Chiem D. N., Foster B. A. & Wasserman S. A. Regulated assembly of the Toll signaling complex drives Drosophila dorsoventral patterning. EMBO J. 23, 100–110 (2004). 14685264 10.1038/sj.emboj.7600033 PMC1271671

[b19] Grosshans J., Schnorrer F. & Nusslein-Volhard C. Oligomerisation of Tube and Pelle leads to nuclear localisation of dorsal. Mech. Dev. 81, 127–138 (1999). 10330490 10.1016/s0925-4773(98)00236-6

[b20] Moynagh P. N. The Pellino family: IRAK E3 ligases with emerging roles in innate immune signalling. Trends Immunol. 30, 33–42 (2009). 19022706 10.1016/j.it.2008.10.001

[b21] Jin W., Chang M. & Sun S. C. Peli: a family of signal-responsive E3 ubiquitin ligases mediating TLR signaling and T-cell tolerance. Cell Mol. Immunol. 9, 113–122 (2012). 22307041 10.1038/cmi.2011.60 PMC4002811

[b22] Kim T. W. *et al.* Pellino 2 is critical for Toll-like receptor/interleukin-1 receptor (TLR/IL-1R)-mediated post-transcriptional control. J. Biol. Chem. 287, 25686–25695 (2012). 22669975 10.1074/jbc.M112.352625 PMC3408172

[b23] Xiao H. *et al.* Pellino 3b negatively regulates interleukin-1-induced TAK1-dependent NF kappaB activation. J. Biol. Chem. 283, 14654–14664 (2008). 18326498 10.1074/jbc.M706931200 PMC2386918

[b24] Jiang Z. *et al.* Pellino 1 is required for interleukin-1 (IL-1)-mediated signaling through its interaction with the IL-1 receptor-associated kinase 4 (IRAK4)-IRAK-tumour necrosis factor receptor-associated factor 6 (TRAF6) complex. J. Biol. Chem. 278, 10952–10956 (2003). 12496252 10.1074/jbc.M212112200

[b25] Haghayeghi A., Sarac A., Czerniecki S., Grosshans J. & Schock F. Pellino enhances innate immunity in Drosophila. Mech. Dev. 127, 301–307 (2010). 20117206 10.1016/j.mod.2010.01.004

[b26] Ferrandon D. *et al.* A drosomycin-GFP reporter transgene reveals a local immune response in Drosophila that is not dependent on the Toll pathway. EMBO J. 17, 1217–1227 (1998). 9482719 10.1093/emboj/17.5.1217 PMC1170470

[b27] Tauszig S., Jouanguy E., Hoffmann J. A. & Imler J. L. Toll-related receptors and the control of antimicrobial peptide expression in Drosophila. Proc. Natl Acad. Sci. USA 97, 10520–10525 (2000). 10973475 10.1073/pnas.180130797 PMC27057

[b28] van Wijk S. J. *et al.* A comprehensive framework of E2-RING E3 interactions of the human ubiquitin-proteasome system. Mol. Syst. Biol. 5, 295 (2009). 19690564 10.1038/msb.2009.55 PMC2736652

[b29] Deshaies R. J. & Joazeiro C. A. RING domain E3 ubiquitin ligases. Annu. Rev. Biochem. 78, 399–434 (2009). 19489725 10.1146/annurev.biochem.78.101807.093809

[b30] Gottar M. *et al.* The Drosophila immune response against Gram-negative bacteria is mediated by a peptidoglycan recognition protein. Nature 416, 640–644 (2002). 11912488 10.1038/nature734

[b31] Schmidt R. L. *et al.* Cleavage of PGRP-LC receptor in the Drosophila IMD pathway in response to live bacterial infection in S2 cells. Self Nonself 2, 125–141 (2011). 22496930 10.4161/self.17882 PMC3323661

[b32] Rus F. *et al.* Ecdysone triggered PGRP-LC expression controls Drosophila innate immunity. EMBO J. 32, 1626–1638 (2013). 23652443 10.1038/emboj.2013.100 PMC3671248

[b33] Wang H., Mu Y. & Chen D. Effective gene silencing in Drosophila ovarian germline by artificial microRNAs. Cell Res. 21, 700–703 (2011). 21423277 10.1038/cr.2011.44 PMC3203660

[b34] Deng L. *et al.* Activation of the IkappaB kinase complex by TRAF6 requires a dimeric ubiquitin-conjugating enzyme complex and a unique polyubiquitin chain. Cell 103, 351–361 (2000). 11057907 10.1016/s0092-8674(00)00126-4

[b35] Huang A. M., Rusch J. & Levine M. An anteroposterior Dorsal gradient in the Drosophila embryo. Genes Dev. 11, 1963–1973 (1997). 9271119 10.1101/gad.11.15.1963 PMC316408

[b36] Braun A., Hoffmann J. A. & Meister M. Analysis of the Drosophila host defence in domino mutant larvae, which are devoid of hemocytes. Proc. Natl Acad. Sci. USA 95, 14337–14342 (1998). 9826701 10.1073/pnas.95.24.14337 PMC24374

[b37] Xia L. *et al.* The Fused/Smurf complex controls the fate of Drosophila germline stem cells by generating a gradient BMP response. Cell 143, 978–990 (2010). 21145463 10.1016/j.cell.2010.11.022

[b38] Glickman M. H. & Ciechanover A. The ubiquitin-proteasome proteolytic pathway: destruction for the sake of construction. Physiol. Rev. 82, 373–428 (2002). 11917093 10.1152/physrev.00027.2001

[b39] Spencer E., Jiang J. & Chen Z. J. Signal-induced ubiquitination of IkappaBalpha by the F-box protein Slimb/beta-TrCP. Genes Dev. 13, 284–294 (1999). 9990853 10.1101/gad.13.3.284 PMC316434

[b40] Shen B., Liu H., Skolnik E. Y. & Manley J. L. Physical and functional interactions between Drosophila TRAF2 and Pelle kinase contribute to Dorsal activation. Proc. Natl Acad. Sci. USA 98, 8596–8601 (2001). 11447260 10.1073/pnas.141235698 PMC37481

[b41] Horng T., Barton G. M., Flavell R. A. & Medzhitov R. The adaptor molecule TIRAP provides signalling specificity for Toll-like receptors. Nature 420, 329–333 (2002). 12447442 10.1038/nature01180

[b42] Yamamoto M. *et al.* Essential role for TIRAP in activation of the signalling cascade shared by TLR2 and TLR4. Nature 420, 324–329 (2002). 12447441 10.1038/nature01182

[b43] Huang H. R., Chen Z. J., Kunes S., Chang G. D. & Maniatis T. Endocytic pathway is required for Drosophila Toll innate immune signaling. Proc. Natl Acad. Sci. USA 107, 8322–8327 (2010). 20404143 10.1073/pnas.1004031107 PMC2889516

[b44] Lund V. K., DeLotto Y. & DeLotto R. Endocytosis is required for Toll signaling and shaping of the Dorsal/NF-kappaB morphogen gradient during Drosophila embryogenesis. Proc. Natl Acad. Sci. USA 107, 18028–18033 (2010). 20921412 10.1073/pnas.1009157107 PMC2964194

[b45] Devergne O., Ghiglione C. & Noselli S. The endocytic control of JAK/STAT signalling in Drosophila. J. Cell Sci. 120, 3457–3464 (2007). 17855388 10.1242/jcs.005926

[b46] Vaccari T., Lu H., Kanwar R., Fortini M. E. & Bilder D. Endosomal entry regulates Notch receptor activation in Drosophila melanogaster. J. Cell Biol. 180, 755–762 (2008). 18299346 10.1083/jcb.200708127 PMC2265571

[b47] Franc N. C., Heitzler P., Ezekowitz R. A. & White K. Requirement for croquemort in phagocytosis of apoptotic cells in Drosophila. Science 284, 1991–1994 (1999). 10373118 10.1126/science.284.5422.1991

[b48] Rubin G. M. & Spradling A. C. Genetic transformation of Drosophila with transposable element vectors. Science 218, 348–353 (1982). 6289436 10.1126/science.6289436

[b49] Rorth P. Gal4 in the Drosophila female germline. Mech. Dev. 78, 113–118 (1998). 9858703 10.1016/s0925-4773(98)00157-9

