# Supplementary material for: Cell-surface localization of Pellino antagonizes Toll-mediated innate immune signalling by controlling MyD88 turnover in Drosophila
Source: Nat Commun. 2014 Mar 17;5:3458. doi: 10.1038/ncomms4458 (PMC3959197; doi:10.1038/ncomms4458)
Supplement: Supplementary Information — Supplementary Figures 1-11 and Supplementary Tables 1-3 [file ncomms4458-s1.pdf]

# Supplementary Figures

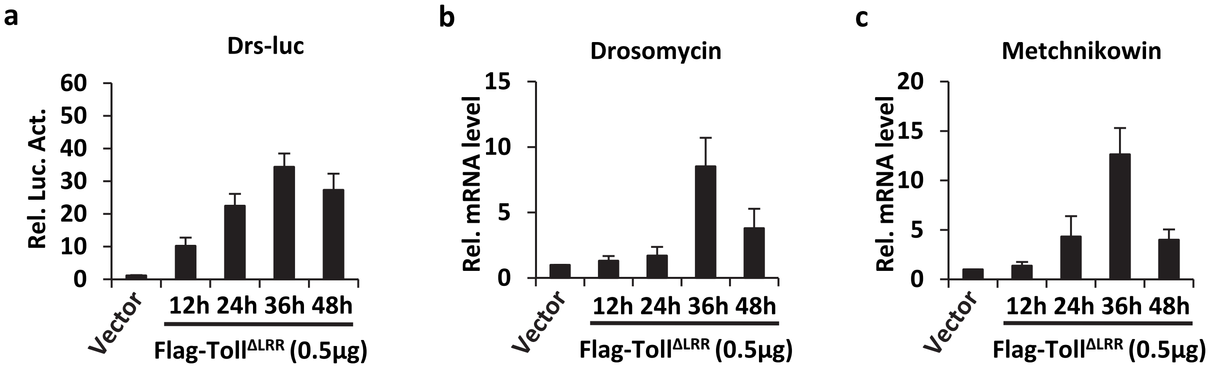


**Supplementary Figure 1. Dynamics of Toll signaling induced by expression of Toll$^{\Delta LRR}$ in S2 cells.**

(**a**) S2 cells were transfected with empty or Toll$^{\Delta LRR}$ constructs together with Drs-luc and Renilla-luc plasmids. Cells were then harvested at different time points and lysed for luciferase reporter assays. Error bars indicate s.d. (n=3). (**b** and **c**) S2 cells were transfected with empty or Toll$^{\Delta LRR}$ plasmids and harvested at various time points. Total RNA was then extracted for quantitative RT-PCR assays to determine transcriptional levels of *drosomycin* (**b**) and *metchnikowin* (**c**). Error bars indicate s.d. (n=3).

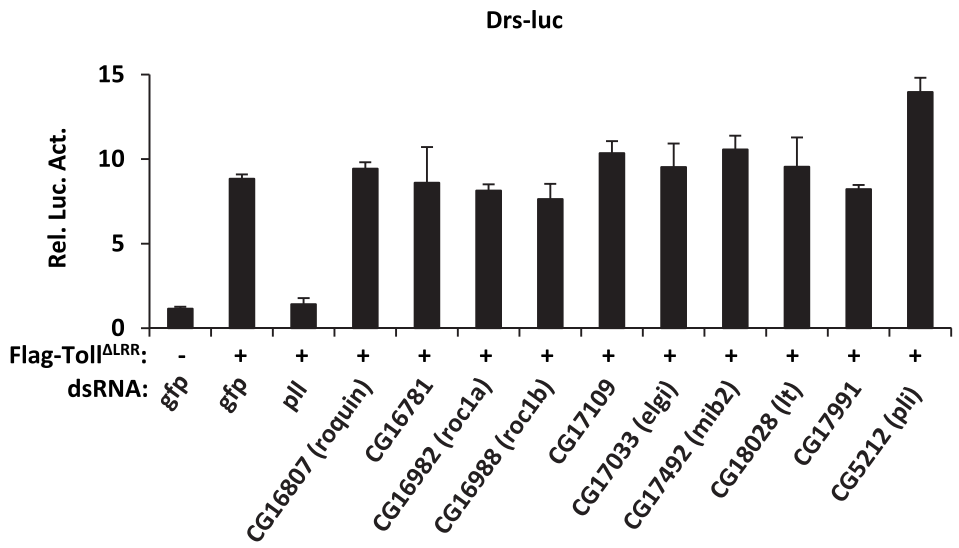


**Supplementary Figure 2. Screening of E3 ligases for searching candidates that potentially regulate Toll signaling.**

S2 cells were treated with dsRNAs targeting for different RING-containing ubiquitin E3 ligases or *gfp* for 48 h, *pelle* dsRNA was used as positive control, then the empty vector or Toll$^{\Delta LRR}$ plasmids were transfected in S2 RNAi cells with Drs-luc and Renilla-luc plasmids. Thirty-six hours after transfection, cells were lysed for luciferase assay. An example of E3 identification is shown. Error bars indicate s.d. (n=3).

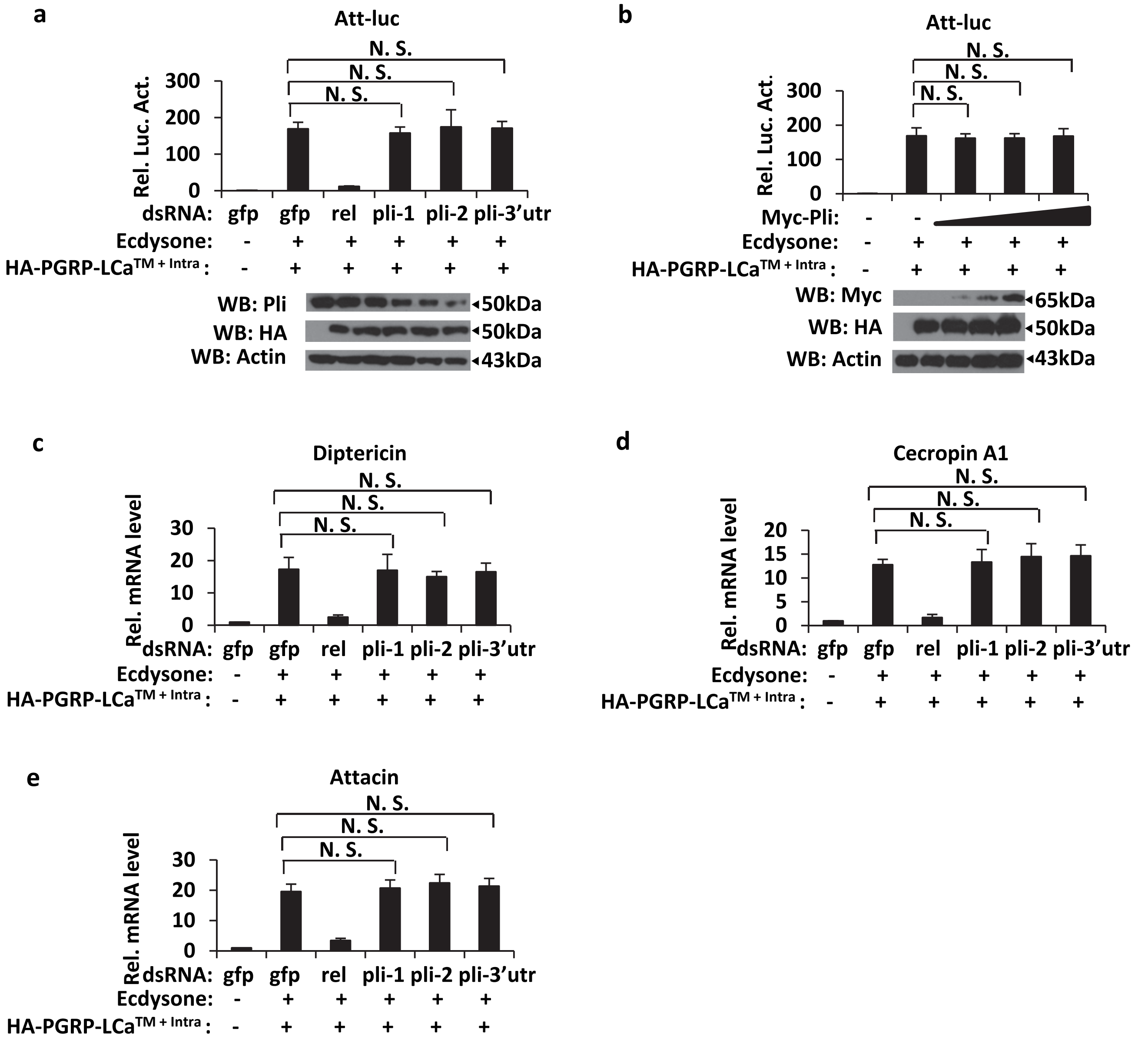


**Supplementary Figure 3. Pellino is dispensable for the IMD signaling.**

(**a**) DsRNAs targeting *gfp*, *relish* (*rel,* positive control) or three different regions of *pellino* were used to treat S2 cells for 48 h, and then empty vector or HA-PGRP-LCa$^{TM+Intra}$ plasmids were transfected into dsRNA-treated S2 cells with Att-luc and Renilla-luc plasmids. Twelve hours after transfection, Ecdysone (the final concentration is 1μM) was added to the cells. Twenty-four hours after Ecdysone treatment, cells were then lysed and subjected to luciferase assays (upper panel) and immunoblotting assays (lower panel). Error bars indicate s.d. (n=3). (**b**) HA-PGRP-LCa$^{TM+Intra}$ and increasing amounts of Myc-Pellino constructs were transfected into S2 cells with Att-luc and Renilla-luc plasmids. After 12 h post-transfection, cells were treated with Ecdysone (1μM) for 24 h, and then lysed for luciferase assay (upper panel), and immunoblotting assays (lower panel). Error bars indicate s.d. (n=3). (**c**-**e**) S2 cells were treated with dsRNAs targeting *pellino, relish* or *gfp*. After 48 h treatment, cells were transfected with DNA vector expressing PGRP-LCa$^{TM+Intra}$. At 12 h post-transfection, cells were treated with Ecdysone (1μM) for 24 h, then total RNA was isolated for quantitative RT-PCR to check transcription levels of *diptericin* (**c**), *cecropinA1*(**d**) and *attacin* (**e**). Error bars indicate s.d. (n=3). For data from **a**-**e**, the two-tailed Student's *t*-test was used to analyze statistical significance. N.S., No Significance, versus control groups.

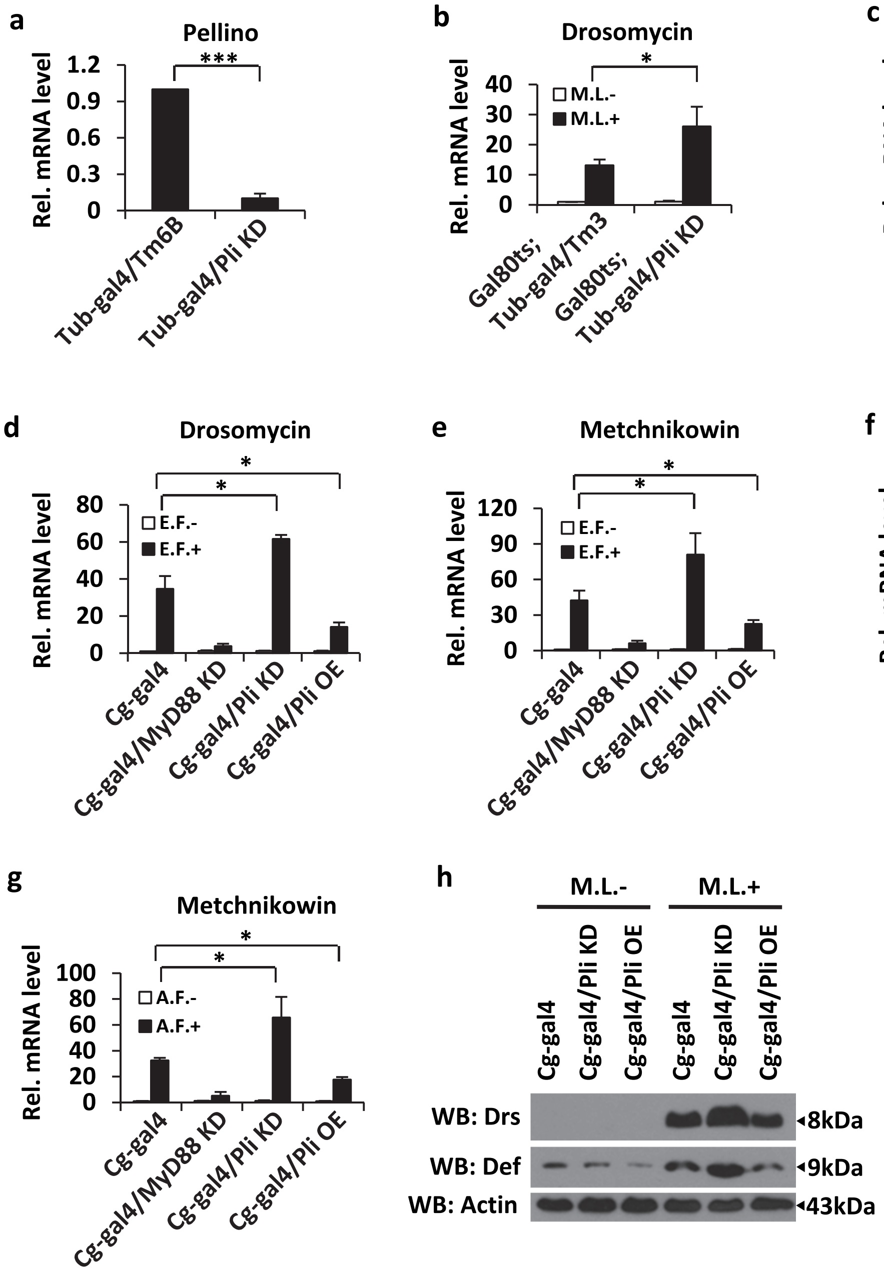


**Supplementary Figure 4. Pellino regulates Toll-mediated immune responses *in vivo.***

(**a**) Crosses were performed in 20°C. *Pellino* knockdown and the control larvae were then shifted to 25°C for 12 h, and larvae were lysed to measure the mRNA level of *pellino*. Error bars indicate s.d. (n=3). (**b** and **c**) The ubiquitous Gal4-Gal80ts driver system was used to control expression of *artmiR-pellino* specifically at the adult stage (seen in Methods and Materials). Six-day-old *pellino* knockdown and control flies were infected with or without *M. luteus* for 12 h and lysed for quantitative RT-PCR analysis to measure mRNA levels of *drosomycin* (**b**) and *metchnikowin* (**c**). Error bars indicate s.d. (n=3). (**d**-**g**) *Pellino* knockdown, *pellino* overexpression, *MyD88* knockdown (positive control) and Cg-gal4 (wild-type control) flies were infected with or without *E. faecalis*(**d** and **e**) or *A. fumigatus* (**f** and **g**) for 12 h and lysed for quantitative RT-PCR analysis to measure mRNA levels of *drosomycin* (**d** and **f**) and *metchnikowin* (**e** and **g**). Error bars indicate s.d. (n=3). (**h**) *Pellino* knockdown, *pellino* overexpression and Cg-gal4 (wild-type control) flies were infected with or without *M. luteus* for 24 h and then lysed for western blot assays to detect the protein levels of Drosomycin and Defensin. Actin is shown as loading control. For data from **a**-**g**, the two-tailed Student's *t*-test was used to analyze statistical significance. * $P < 0.05$, ** $P < 0.01$, *** $P < 0.001$, versus control groups.

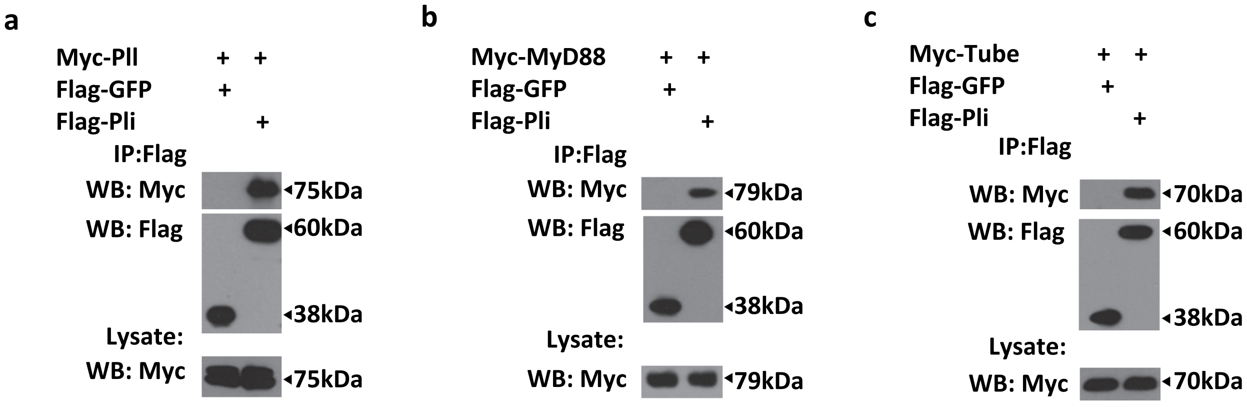


**Supplementary Figure 5. Pellino interacts with Pelle, Tube and MyD88.**

(**a**-**c**) S2 cells were transfected with combinations of expression plasmids as indicated. Forty-eight hours after transfection, cell lysates were prepared, immunoprecipitated with anti-Flag beads, followed by immunoblot analysis with the indicated antibodies. Levels of Myc-Pelle expression in whole-cell lysates are shown in bottom panel.

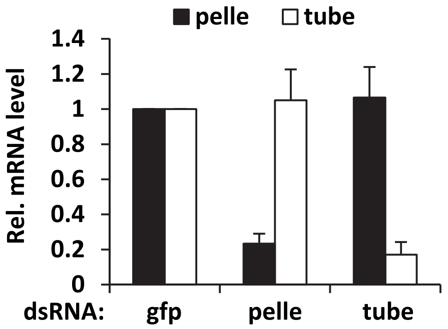


**Supplementary Figure 6. Pelle and tube were knockdown by dsRNA efficiently.**

S2 cells were treated with dsRNA targeting *gfp*, *pelle* or *tube* for 48 h, and then total RNA was extracted and subjected to quantitative RT-PCR analysis. Error bars indicate s.d. (n=3).

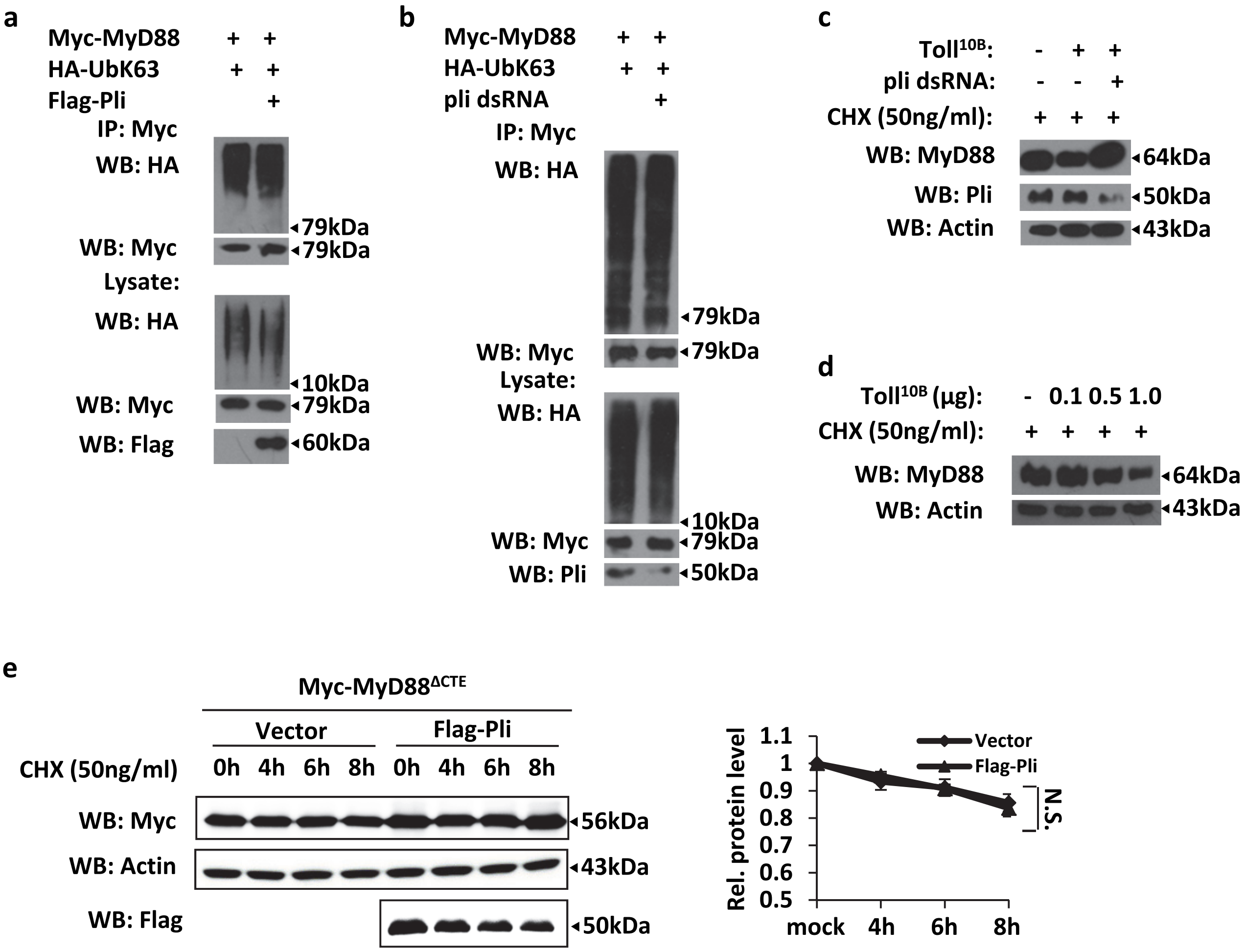


**Supplementary Figure 7. MyD88 ubiquitination is regulated by Pellino through K-48 linkage, and depends on the CTE domain of MyD88.**

(**a** and **b**) S2 cells were transfected with combinations of expression plasmids as indicated (**a**), or cells were also pretreated with *pellino* dsRNA (**b**). Cell lysates were then used to perform immunoprecipitation experiments with anti-Myc beads, followed by immunoblot analysis with anti-HA antibodies to show K63-likaged ubiquitination of MyD88. (**c**) S2 cells were transfected with $Toll^{10B}$ expression plasmids as indicated. Thirty-six hours after transfection, cells were treated with CHX (50 ng/ml) for 8 h, and then were lysed to perform western blotting assays. (**d**) $Toll^{10B}$ expression plasmids were transfected into S2 cells, which were pretreated with or without *pellino* dsRNA for 48 h as indicated. Thirty-six hours after transfection, cells were then treated with CHX (50 ng/ml) for 8 h and lysed to perform western blotting assays. (**e**) S2 cells were transfected with expression constructs as indicated, and after 48 h post-transfection, cells were treated with CHX (50 ng/ml) for different times, followed by immunoblotting to check $MyD88^{\Delta CTE}$ expression levels (left panel). Densitometry analysis to quantify $MyD88^{\Delta CTE}$ expression is shown in the right panel. Error bars indicate s.d. (n=3). For data from **e**, the LogRank test was used to analyze the variance of the protein stability between the two groups. N.S., No Significance, versus control groups.

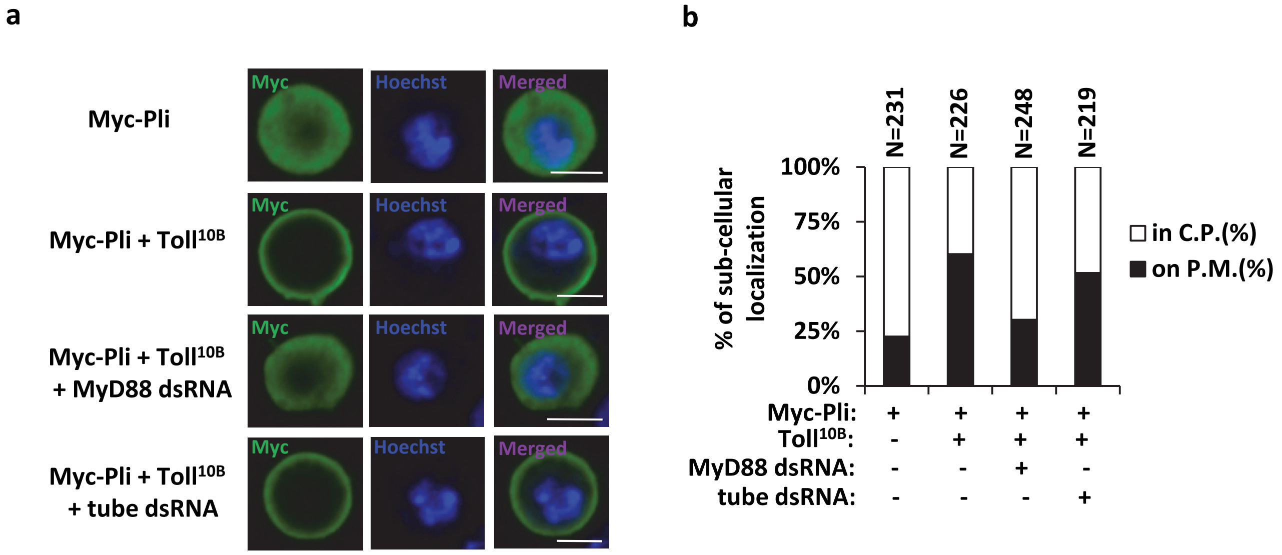


**Supplementary Figure 8. Toll signaling induced by expression of Toll$^{10B}$ promotes Pellino cell-surface localization in a MyD88 dependent manner.**

(**a**) S2 cells were transfected with combinations of expression plasmids as indicated, or cells were also pretreated with *MyD88 or Tube* dsRNA. Cells were further stained with anti-Myc antibody and Hoechst, and imaged by confocal microscopy. Scale bars, 10μm. (**b**) Statistical assays of plasma membrane (P.M.) or cytoplasmic (C.P.) localization of Pellino in cell samples from panel (**a**).

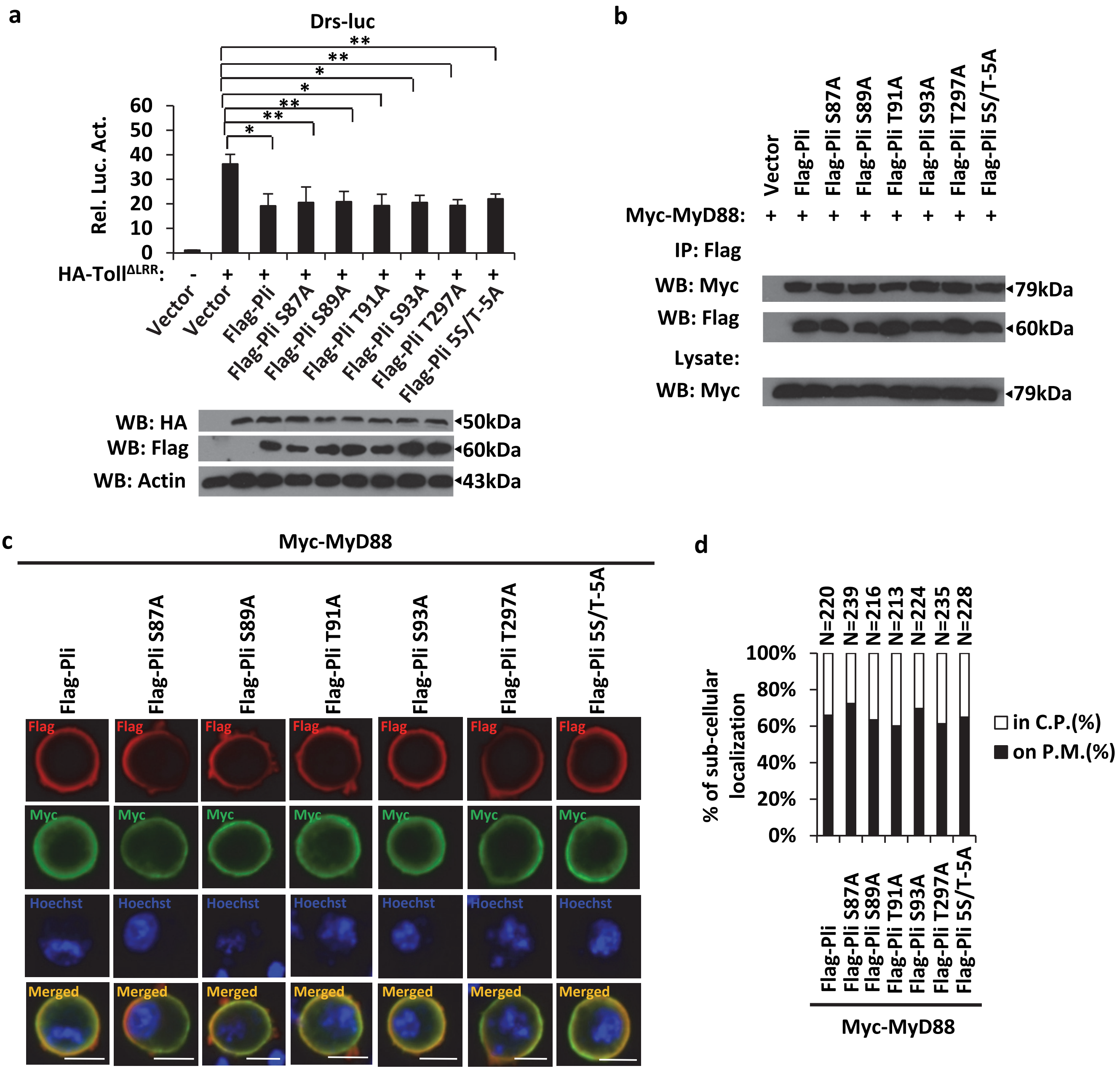


**Supplementary Figure 9. Mutations of potential phosphorylation sites in Pellino have no effect on its function.**

(**a**) Different potential phosphorylation sites mutations of Pellino expressing vectors were generated to detect their functions in regulating Toll immune signaling. S2 cells were transfected with empty vectors or expression plasmids as indicated. Thirty-six hours after transfection, cells were lysed for reporter assays (upper panel), and immunoblotting assays performed with the indicated antibodies (lower panel). Error bars indicate s.d. (n=3). Flag-Pli 5S/T-5A: Flag-Pellino containing five S or T to A mutations at S87, S89, T91, S93, T297 site. (**b**) S2 cells were transfected with combinations of expression plasmids as indicated. Forty-eight hours after transfection, cell lysates were prepared, immunoprecipitated with anti-Flag beads, followed by immunoblot analysis with the indicated antibodies. Expression levels of Myc-MyD88 in whole-cell lysates are shown in bottom panel. (**c** and **d**) S2 cells were transfected with Myc-MyD88 in combination with wild-type or different point mutation of Flag-Pellino expression plasmids. Thirty-six hours later, cells were fixed and stained with Hoechst, anti-Flag and anti-Myc antibodies, then imaged by confocal microscopy(**c**). Scale bars, 10μm. (**d**) Statistical assays of plasma membrane (P.M.) or cytoplasmic (C.P.) localization of Pellino in cell samples from panel (**c**). For data from **a**, the two-tailed Student's *t*-test was used to analyze statistical significance. * $P < 0.05$, ** $P < 0.01$, versus control groups.

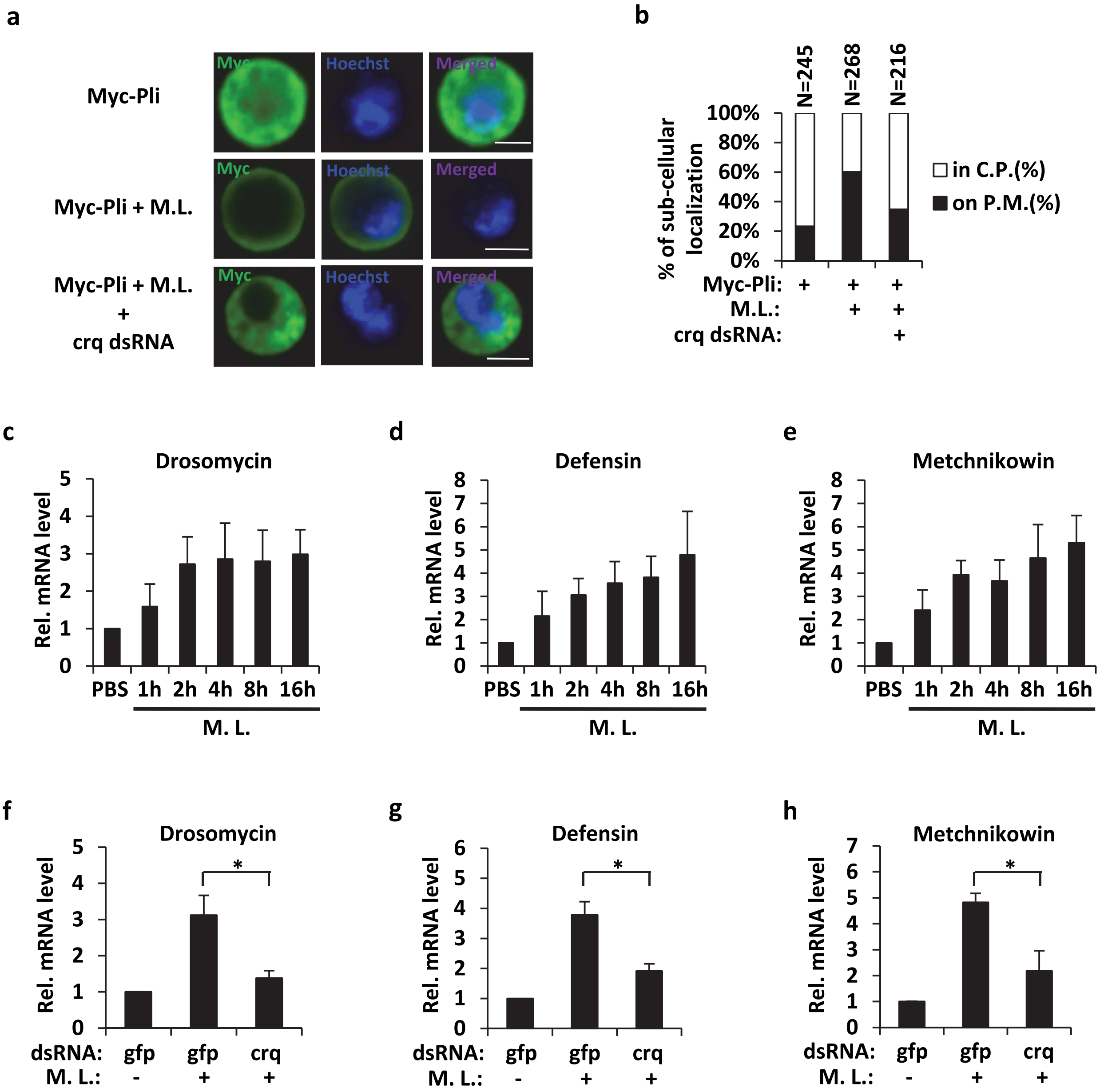


**Supplementary Figure 10.** ***Croquemort*** **potentially regulates the cell-surface accumulation of Pellino upon** ***M. Luteus*** **stimulation.**

(**a**) S2 cells were transfected with combinations of expressing vectors as indicated, or cells were also pretreated with *croquemort (crq)* dsRNA. Twenty-four hours after transfection, cells were treated with *M. Luteus* for 12 h, then stained with anti-Myc antibody and Hoechst, and imaged by confocal microscopy. Scale bars,10μm. (**b**) Statistical assays of plasma membrane (P.M.) or cytoplasmic (C.P.) localization of Pellino in cell samples from panel (**a**). (**c**-**e**) S2 cells were treated with or without *M. Luteus* and then harvested at different time points as indicated. Total RNA was extracted and subjected to quantitative RT-PCR analysis. Error bars indicate s.d. (n=3). (**f**-**h**) S2 cells were treated with *gfp* or *crq* dsRNAs for 48 h and then treated with or without *M. Luteus* for 12 h as indicated. Total RNA was extracted and subjected to quantitative RT-PCR analysis. Error bars indicate s.d. (n=3). For data from **f**-**h**, the two-tailed Student's *t*-test was used to analyze statistical significance. * $P < 0.05$, versus control groups.

Figure 1a:

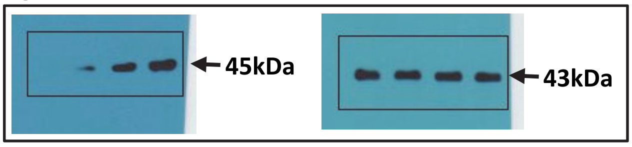


Figure 1b:

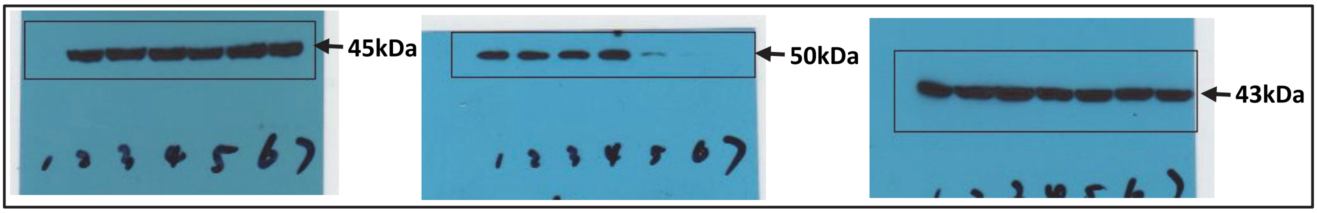


Figure 1f:

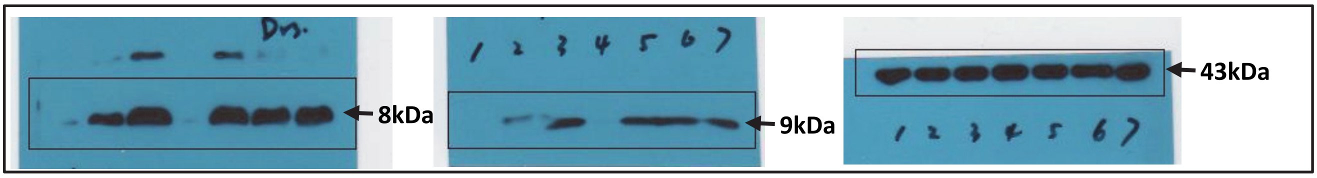


Figure 1g:

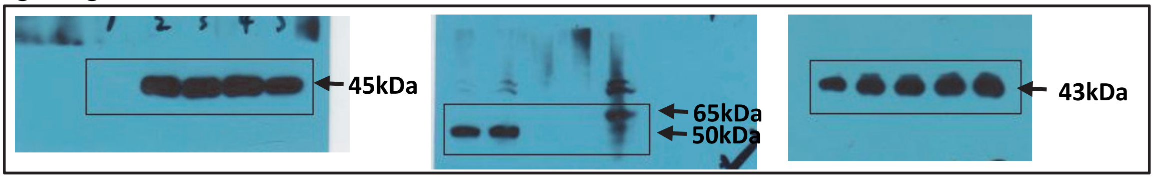


Figure 1h:

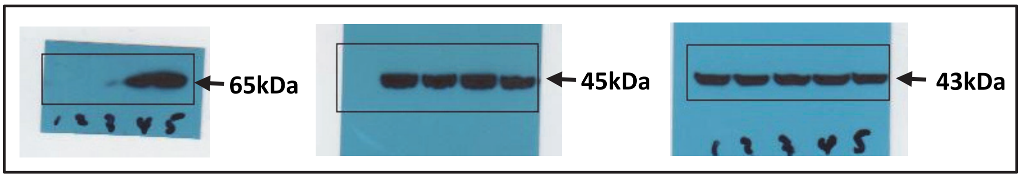


Figure 2a:

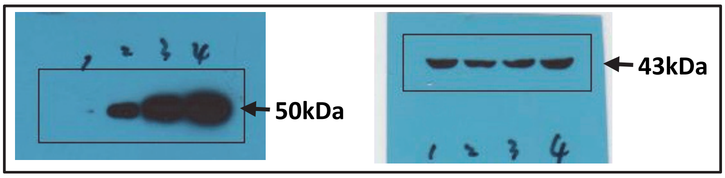


Figure 2b:

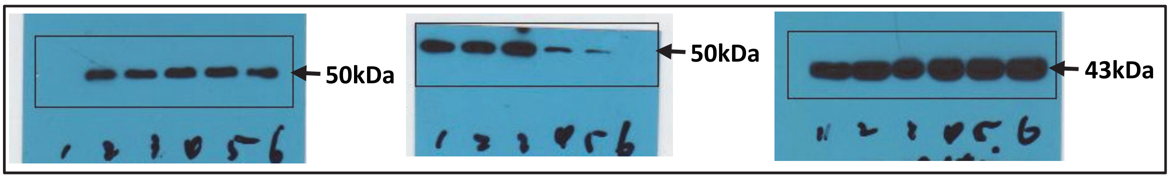


Figure 2c:

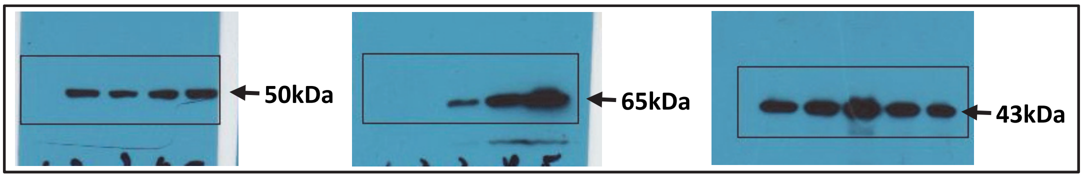


**Supplementary Figure 11. Original western blots in main and supplementary figures. Panels corresponding to the figures are indicated.**

Figure 3c:

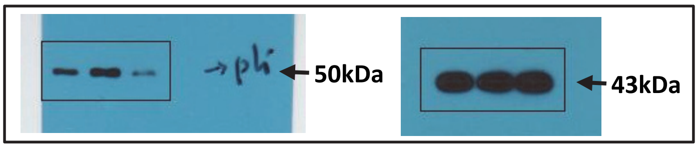


Figure 4a:

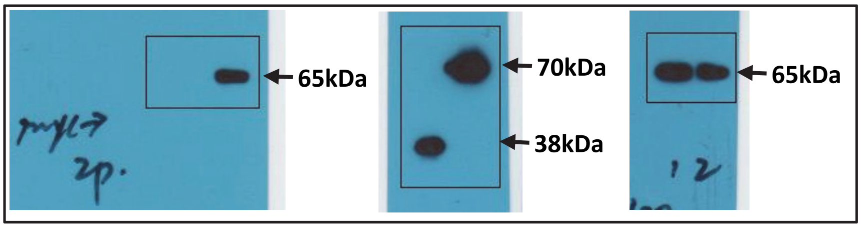


Figure 4b:

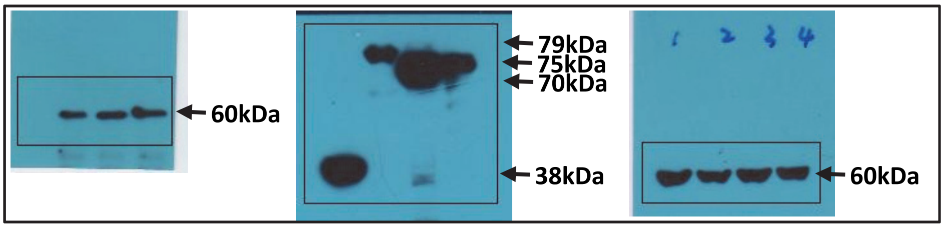


Figure 4c:

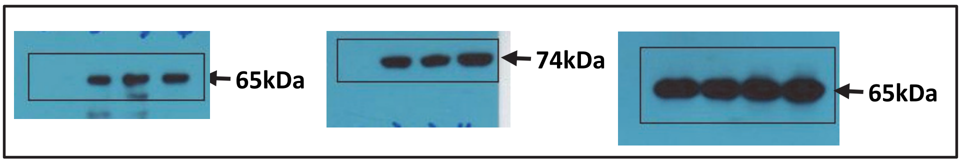


Figure 4d:

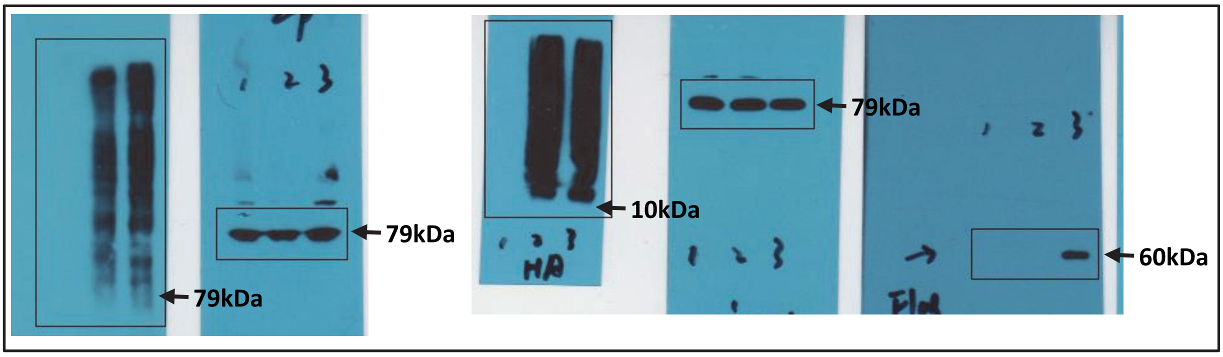


Figure 4e:

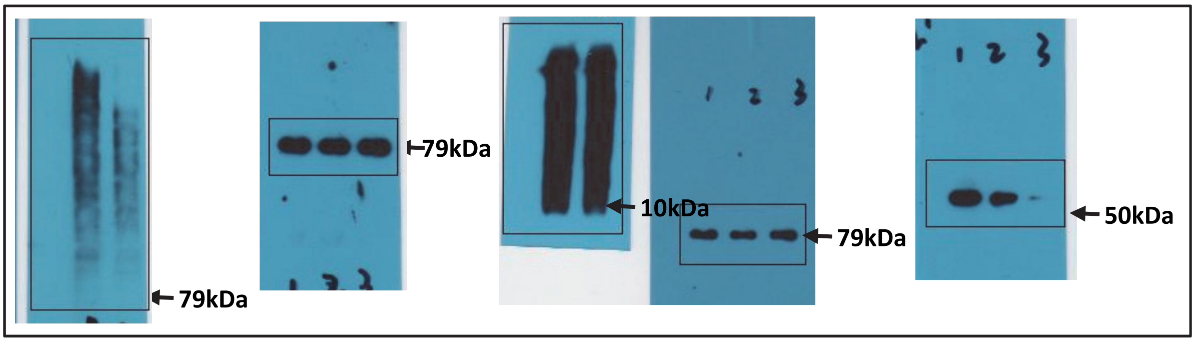


**Supplementary Figure 11 continued.**

Figure 4f:

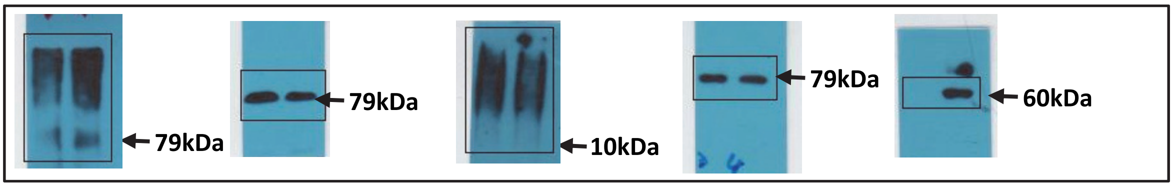


Figure 4g:

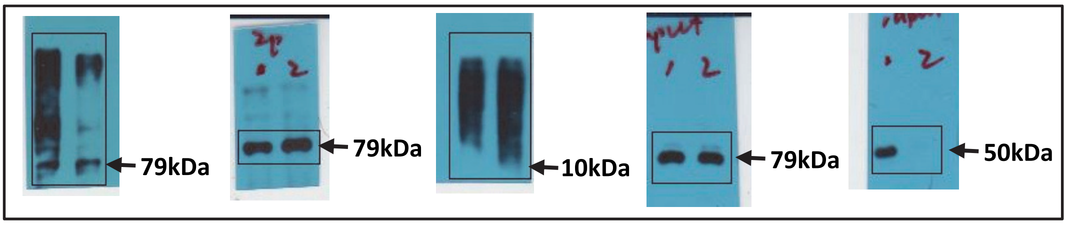


Figure 4h:

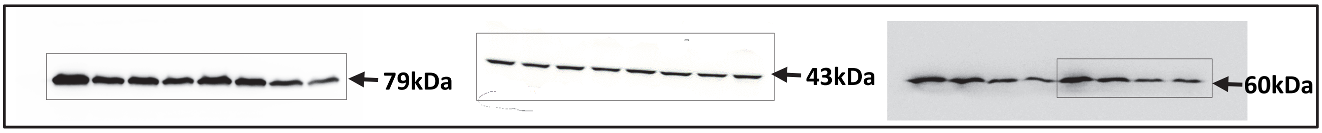


Figure 4i:

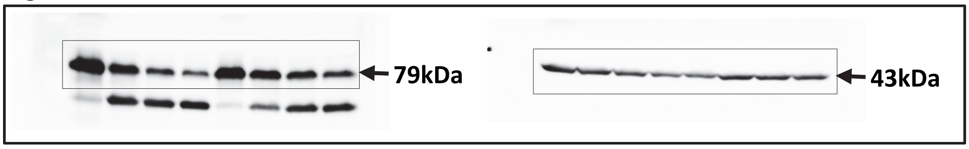


Figure 5f:

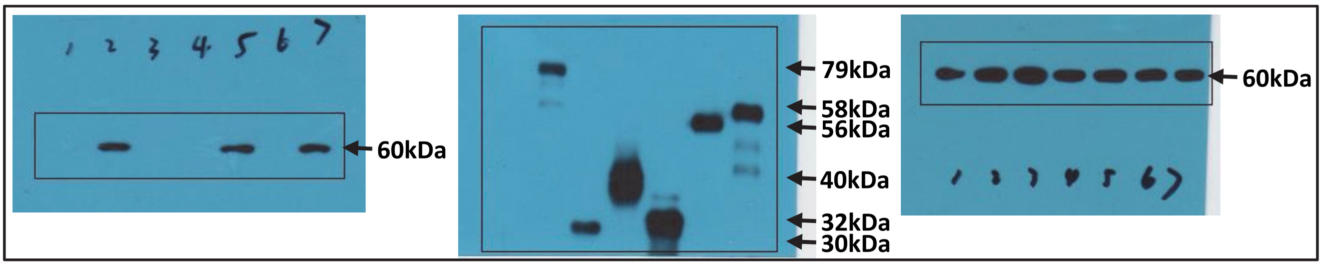


Figure 6a:

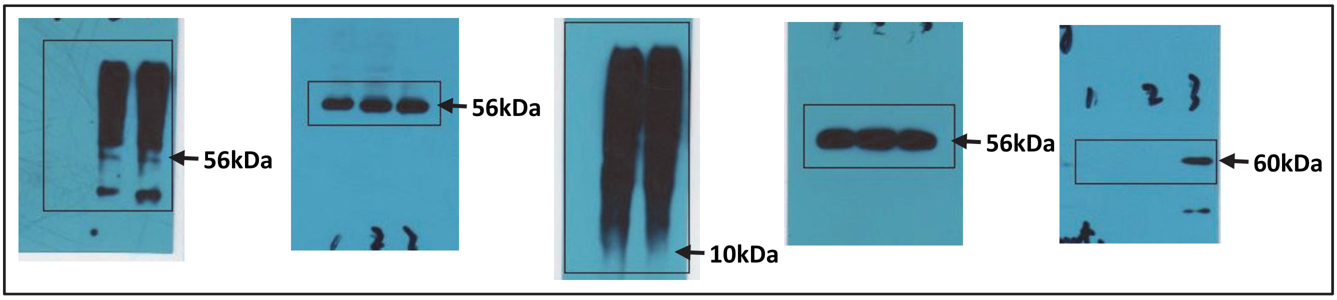


Figure 6b:

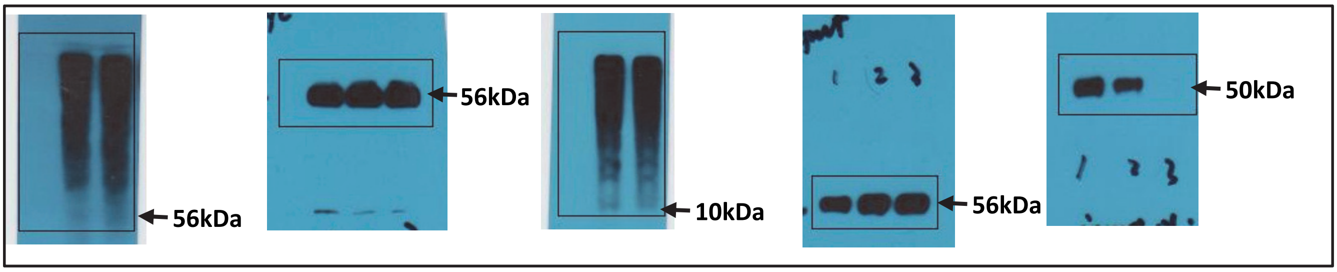


**Supplementary Figure 11 continued.**

Figure S3a:

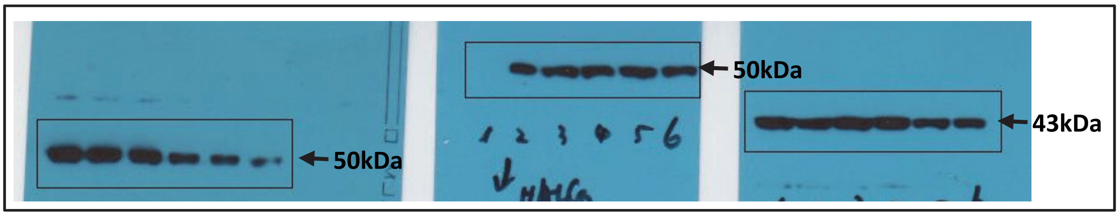


Figure S3b:

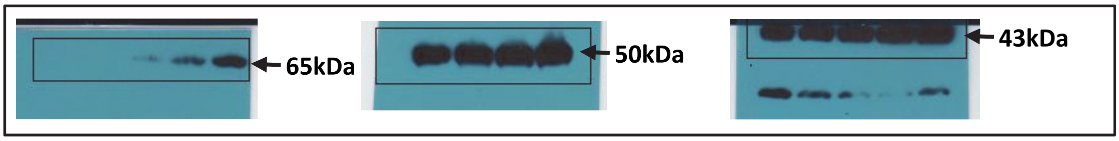


Figure S4h:

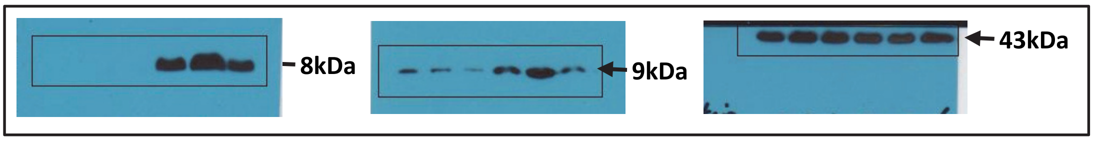


Figure S5a:

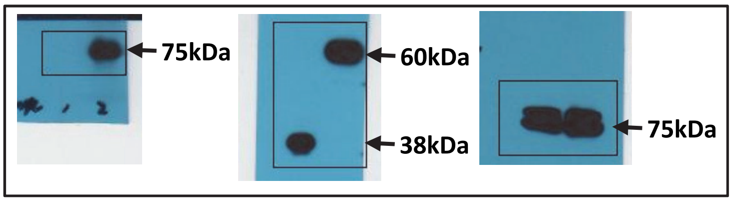


Figure S5b:

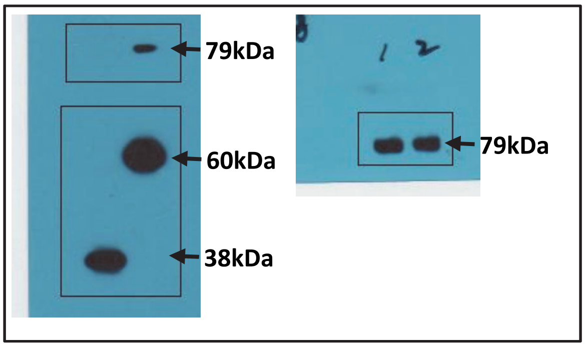


Figure S5c:

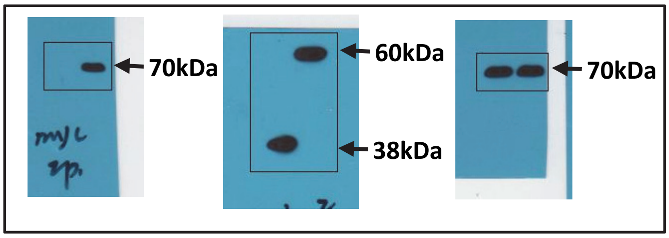


Figure S7a:

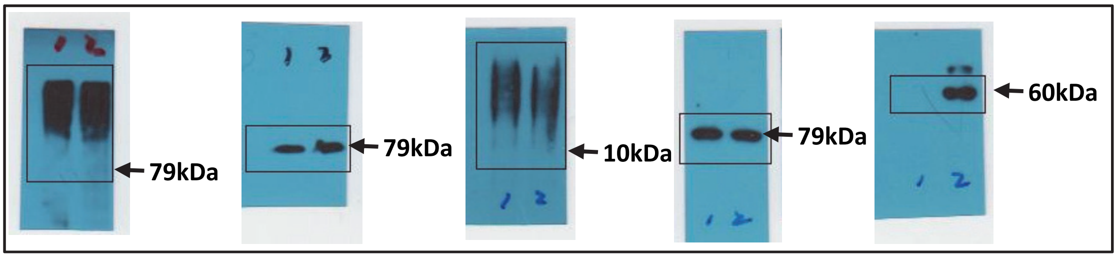


**Supplementary Figure 11 continued.**

Figure S7b:

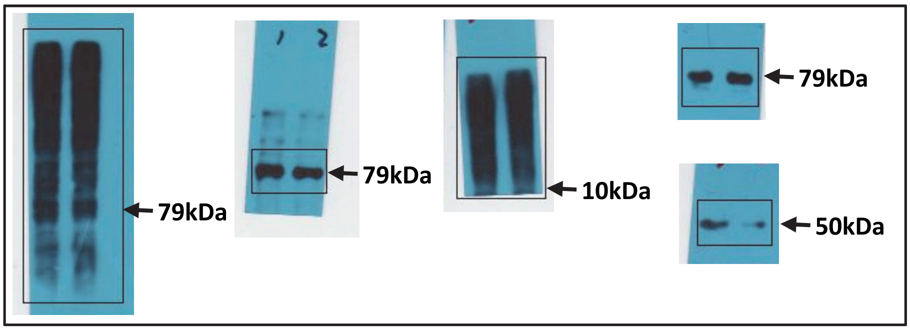


Figure S7c:

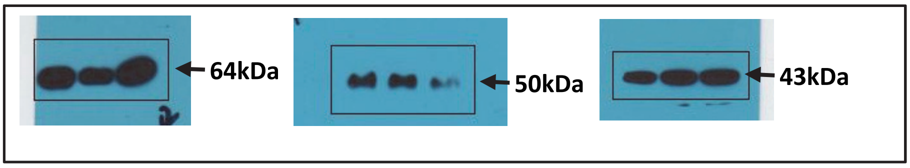


Figure S7d:

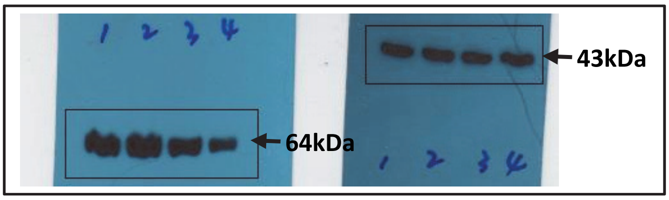


Figure S7e:

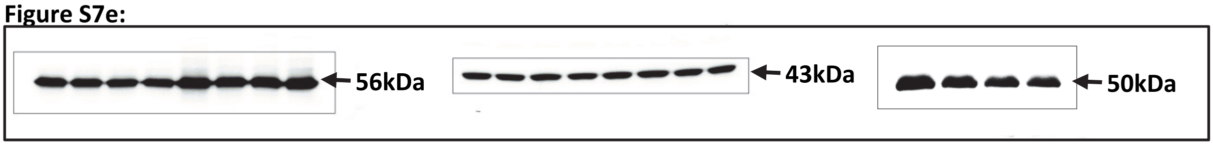


Figure S9a:

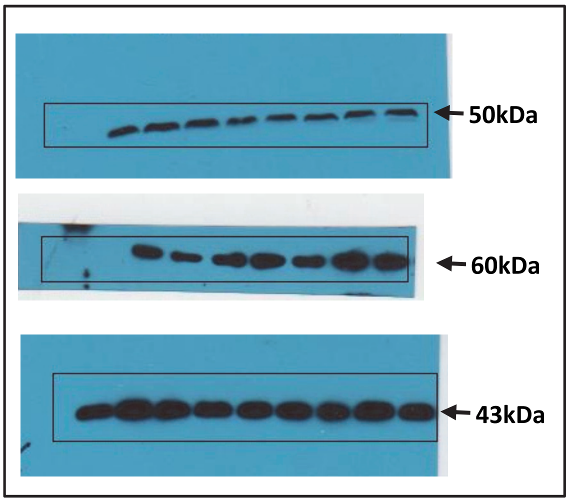


Figure S9b:

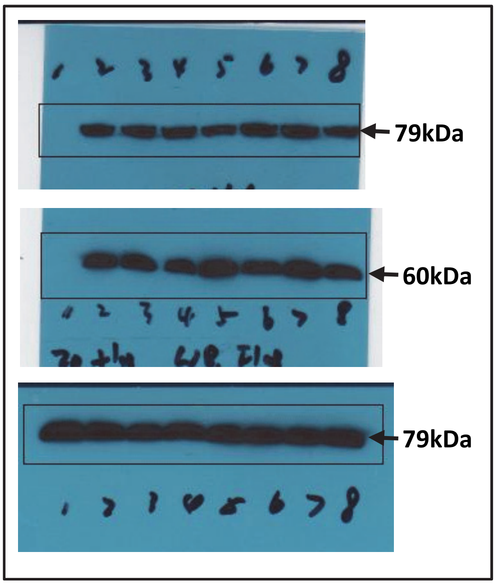


**Supplementary Figure 11 continued.**

**Supplementary Table 1. Primers used for transgene vector construction:**

| Name: | Sequence: |
|---|---|
| Pellino-OE-s | ATACTCGAGATGGTGAAACGCACCGACGGC |
| Pellino-OE-as | TATGCTAGCTTAATCTAGATTATCCTGGAAT |
| Pellino-KD-1-s | CTAGCAGTCGCAATGATCTGCAAGCAGGATAGTTATATTCAAGCATATGCTGCTTGCTGATCATTG CGGCG |
| Pellino-KD-1-as | AATTCGCCGCAATGATCAGCAAGCAGCATATGCTTGAATATAACTATCCTGCTTGCAGATCATTGC GACTG |
| Pellino-KD-2-s | CTAGCAGTCGTTGAACCTTATTATTCGGATAGTTATATTCAAGCATATGCGAATAATTAGGTTCAA CGGCG |
| Pellino-KD-2-as | AATTCGCCGTTGAACCTAATTATTCGCATATGCTTGAATATAACTATCCGAATAATAAGGTTCAAC GACTG |

**Supplementary Table 2. Primers used for dsRNA synthesis:**

| Name: | Sequence: |
|---|---|
| gfp-F | CTCACTATAGGGAGAAGCAAGGGCGAGGAGCTGTT |
| gfp-R | CTCACTATAGGGAGAGGTAGTGGTTGTCGGGCAGC |
| cactus-F | CTCACTATAGGGAGACCACGTCCACTGATCCCGAAATAC |
| cactus-R | CTCACTATAGGGAGAAAAGCAGCGGAGGCAGCAACAAAG |
| pll-F | CTCACTATAGGGAGACACAAGTACATACCGAGGAG |
| pll-R | CTCACTATAGGGAGAGCTGATGCTAAACCGCTGCT |
| pli-1-F | CTCACTATAGGGAGATCACGCAACCAGGCGGTAATC |
| pli-1-R | CTCACTATAGGGAGAAGATCAATAAGTGTGCCATCC |
| pli-2-F | CTCACTATAGGGAGACCGGCTTTGATTCGAGCAGAA |
| pli-2-R | CTCACTATAGGGAGAGGCCCTGGACATGGCCACAGT |
| pli-3’utr-F | CTCACTATAGGGAGAGAGTGCTCCGTGGGCGGCGAC |
| pli-3’utr-R | CTCACTATAGGGAGATTGACAGTTTTTTCAGTCGCC |
| rel-F | CTCACTATAGGGAGAAGCAACGCCGAAACTAACG |
| rel-R | CTCACTATAGGGAGAAGTACTACGACCTGGACAATG |
| tube-F | CTCACTATAGGGAGAGAGTGGAAGACC |
| tube-R | CTCACTATAGGGAGACTGAATTTGTTC |
| MyD88-F | CTCACTATAGGGAGAATCGATCATGCC |
| MyD88-R | CTCACTATAGGGAGACTTGTCGTTGAT |
| crq-F | ACTATAGGGAGA GGTTACCAGGAT |
| crq-R | ACTATAGGGAGA TTGCACTCCACG |

**Supplementary Table 3. Primers used for quantitative RT-PCR:**

| **Name:** | **Sequence:** |
|---|---|
| pli-F | ATGGGCTTGGAACCAGCCTTCTAT |
| pli-R | TGAAATCCGTTGGTACCGTGAGGT |
| drs-F | ATCCTGAAGTGCTGGTGCGAAGGAG |
| drs-R | ACGTTCATGCTAATTGCTCATGG |
| metch-F | CAGTGCTGGCAGAGCCTCAT |
| metch-R | CAACCATAAATTGGACCCGGTCTTGGTG |
| def-F | CCACATGCGACCTACTCTCCA |
| def-R | GACAAGAACGCAGACGGCCTTGC |
| cecA1-F | TTTCGTCGCTCTCATTCTGG |
| cecA1-R | GACAATCCCACCCAGCTTCCCGATTGC |
| dpt-F | TTTGCAGTCCAGGGTCACCA |
| dpt-R | CACGAGCCTCCATTCAGTCCAATCTCGG |
| att-F | CACCAGATCCTAATCGTGGCCCTGGG |
| att-R | ACGCGAATGGGTCCTGTTGT |
| rp49-F | CACGATAGCATACAGGCCCAAGATCGG |
| rp49-R | GCCATTTGTGCGACAGCTTAG |
| pll-F | TGGACGCTGTGGTGGAAGTGAATA |
| pll-R | TGAACACCTCCAACAGCACAATGC |
| tube-F | AACTCTCGACCAAATCACGCTCCA |
| tube-R | AAGGTCTCCCTGCTGCCTTTACTT |
